# Transcriptomics Unveil Canonical and Non-Canonical Heat Shock-Induced Pathways in Human Cell Lines

**DOI:** 10.3390/ijms26031057

**Published:** 2025-01-26

**Authors:** Andrew Reinschmidt, Luis Solano, Yonny Chavez, William Drew Hulsy, Nikolas Nikolaidis

**Affiliations:** Department of Biological Science, Center for Applied Biotechnology Studies, and Center for Computational and Applied Mathematics, California State University Fullerton, Fullerton, CA 92831, USA; areinschmidt4@csu.fullerton.edu (A.R.); lesolano@uci.edu (L.S.); yonny@csu.fullerton.edu (Y.C.); wdhulsy@csu.fullerton.edu (W.D.H.)

**Keywords:** heat shock response (HSR), RNA sequencing, cellular stress response (CSR)

## Abstract

The cellular stress response (CSR) is a conserved mechanism that protects cells from -environmental and physiological stressors. The heat shock response (HSR), a critical component of the CSR, utilizes molecular chaperones to mitigate proteotoxic stress caused by elevated temperatures. We hypothesized that while the canonical HSR pathways are conserved across cell types, specific cell lines may exhibit unique transcriptional responses to heat shock. To test this, we compared the transcriptomic responses of HEK293, HepG2, and HeLa cells under control conditions immediately following heat shock and after an 8-h recovery period. RNA sequencing revealed the conserved activation of canonical HSR pathways, including the unfolded protein response, alongside the -enrichment of the non-canonical “Receptor Ligand Activity” pathway across all cell lines. Cell-line-specific variations were observed, with HepG2 cells exhibiting significantly higher ex-pression levels of certain genes compared to other cell lines under stress conditions, as well as greater fold changes in gene expression relative to its control conditions. Validation by qPCR confirmed the activation of key genes within the “Receptor Ligand Activity” pathway across time points. These findings provide insights into conserved and context-specific aspects of the HSR, contributing to a more comprehensive understanding of stress response mechanisms across mammalian cells.

## 1. Introduction

The cellular stress response (CSR) is a critical survival mechanism that helps cells cope with environmental stressors, and the heat shock response (HSR) is one of its most essential components [1,2,3,4,5,6,7]. The HSR is primarily activated by temperature increases and other stressors, triggering the activation of heat shock factors (HSFs), especially HSF1, which then drive the expression of heat shock proteins (HSPs). These molecular chaperones, including HSP70 and HSP90, facilitate protein homeostasis by refolding misfolded proteins, preventing aggregation, and modulating apoptosis [3,4,5,8,9,10,11,12]. While the canonical HSR is well-characterized, the full scope of its regulatory mechanisms and potential adaptations to different cellular contexts still need to be understood [13]. In particular, non-canonical HSR pathways and their roles in cellular stress adaptation are less explored [14], despite their potential significance in various disease processes.

In cancer, the HSR’s molecular machinery is hijacked for cellular survival, enabling tumor cells to withstand the proteotoxic stress generated by rapid proliferation, hypoxia, and therapeutic interventions. Cancer cells frequently upregulate key HSR components, including HSF1, to maintain proteostasis and resist apoptosis induced by therapies [15,16,17,18]. While much is known about the activation of canonical HSR pathways in cancer, a significant gap remains in understanding how these pathways are modulated in cancer cells and whether non-canonical HSR pathways contribute to the enhanced stress resistance observed in tumors. Current research broadly focuses on the well-established roles of HSPs and HSF1. Still, the complexity of the HSR—particularly its dynamic crosstalk with other stress response networks like the unfolded protein response (UPR) and DNA damage response (DDR)—and how these interactions regulate stress resistance in tumors remain underexplored [1,2,3,4,5,6,7,8,9,10,11,12].

This study aims to fill these gaps by conducting a comprehensive RNA sequencing (RNA-seq) analysis comparing cancerous (HeLa, HepG2) and non-cancerous (HEK293) cell lines exposed to heat stress. By comparing these cell lines, we aim to identify both conserved and cell-specific mechanisms regulating the HSR, with particular emphasis on uncovering non-canonical pathways that may play a significant role in cellular stress adaptation. We hypothesize that, while canonical HSR pathways are activated across all cell types, cancer cells will exhibit unique regulatory modifications and interactions, particularly involving non-canonical HSR pathways. These candidate pathways may contribute to enhanced survival and therapy resistance phenotypes.

By identifying these novel non-canonical HSR targets, we anticipate uncovering new insights into how cells adapt to stress, particularly in the context of cancer. Moreover, our study will provide a deeper understanding of the full spectrum of HSR mechanisms, including those understudied. This research has broad implications not only for cancer biology but also for understanding the role of stress responses in a variety of other diseases, including neurodegenerative disorders and aging, where similar proteotoxic stress is a hallmark [19,20]. By uncovering the molecular mechanisms driving non-canonical HSR pathways, this work can potentially inform the development of novel therapeutic strategies that target stress responses, providing new avenues for treating cancer and other stress-related diseases.

## 2. Results

### 2.1. Principal Component Analysis

**Figure 1:**
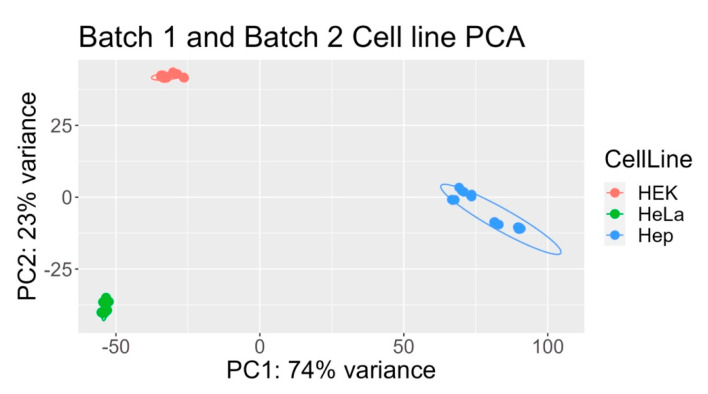
Principal component analysis (PCA) of variance-stabilizing transformed (VST) counts reveals that the primary source of variance in the entire dataset is the cell line type, regardless of batch or heat shock treatment. PCA reduces high-dimensional gene expression data to principal components, visually representing clustering patterns based on sample similarities. Each point represents a sample, with closer proximity indicating higher similarity.

To identify key drivers of variance in the transcriptomic dataset, we conducted a principal component analysis (PCA) using VST-normalized read counts of the entire dataset [all three cell lines (HEK293, HepG2, and HeLa) under three conditions (control, 0 h recovery after heat shock, and 8 h recovery after heat shock) across two experimental batches (Batch 1 and Batch 2)]. PCA results of the entire dataset reveal that samples primarily cluster by cell line, regardless of heat shock condition or batch, indicating that cell line type is the most significant source of variance in gene expression (Figure 1).

Further PCA analyses on individual cell lines show that within-cell line variance is predominantly driven by experimental batch effects (Supplementary Figure S1). These findings necessitated batch-specific analyses to mitigate confounding effects.

**Figure 2:**
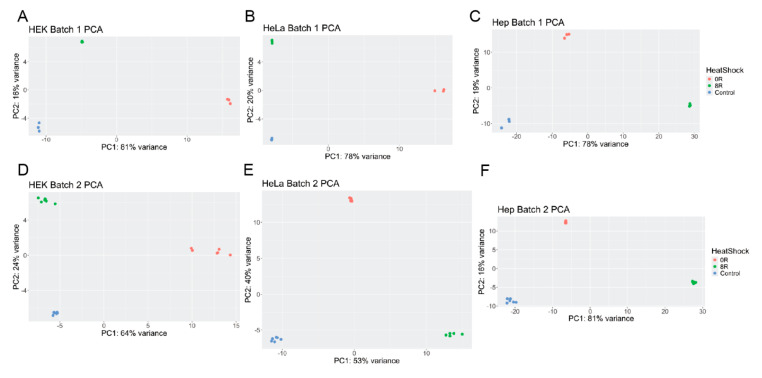
PCA of VST--normalized counts, stratified by batch and cell line, highlights clustering based on heat shock conditions. Individual PCA plots for HEK293 (**A**,**D**), HeLa (**B**,**E**), and HepG2 (**C**,**F**) show distinct groupings between heat-shocked and control samples, indicating a robust transcriptional response to heat shock across all cell lines. The separation along principal components reflects the contribution of gene expression differences induced by heat shock and recovery time.

Figure 2 stratifies the analysis by cell line and batch, showing that heat shock conditions (0 h and 8 h recovery) explain most of the variance within each cell line. When analyzing data separated by cell line and batch, PCA revealed that heat shock conditions significantly contributed to gene expression variance (Figure 2). For the HEK293 cell line, heat shock (0 h recovery) explained 81% of the variance in batch 1 (and 65% in batch 2; Figure 2A,D), emphasizing a robust transcriptional response immediately following stress. For HeLa cells, heat shock accounted for 77% of the variance for batch 1, while recovery from heat shock could explain 53% of the variance for batch 2 (Figure 2B,E). HepG2 cells displayed a distinct pattern: most of the variance was explained by recovery from heat shock (8 h), with PC1 capturing 80% of the variance in batch 1 (and 78% in batch 2; Figure 2C,F). The observed pattern highlights cell-line-specific differences in the heat shock response (HSR). These findings collectively establish that cell type, batch, and heat shock are the principal drivers of transcriptomic variance while revealing unique stress response and recovery dynamics across cell lines.

### 2.2. Differential Gene Expression Analysis

We performed a differential gene expression (DGE) analysis to evaluate transcriptional changes across conditions for each cell line. The number of differentially expressed genes (DEGs) varied across conditions and batches, reflecting biological and technical factors. Differences in cell line-specific responses to heat shock, recovery dynamics, and batch-specific technical variability likely contributed to these discrepancies. Additionally, the increased sample size in Batch 2 provided greater statistical power to detect smaller changes in gene expression.

**Figure 3:**
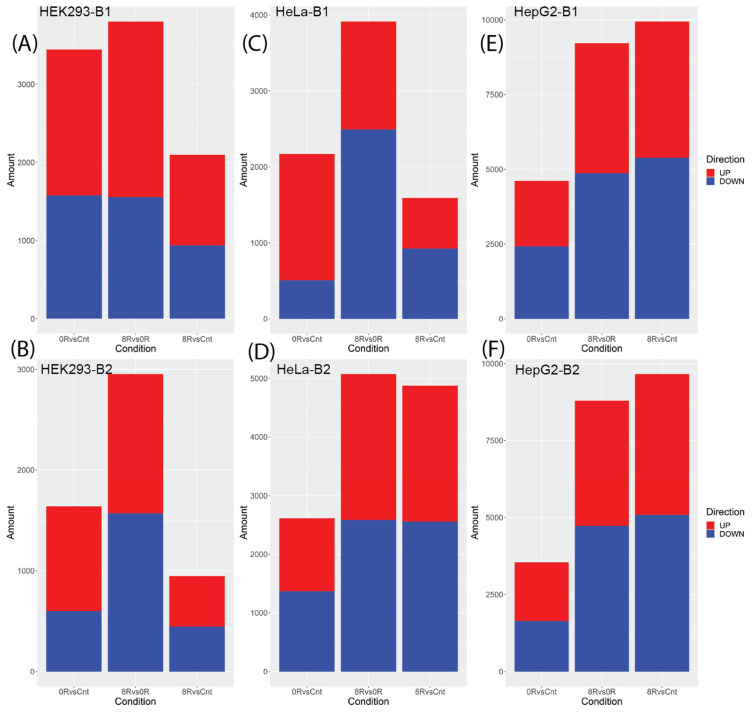
The Number of DEGs varies by Cell Line and Batch. Histogram depicting the number of DEGs (|log2 fold change| > 0.5; adjusted *p*-value < 0.05) in HEK293 (**A**,**B**), HeLa (**C**,**D**), and HepG2 (**E**,**F**) cell lines during heat shock for batch 1 (**top rows**) and batch 2 (**bottom rows**). The height of each bar represents the count of upregulated and downregulated genes, emphasizing the scale of transcriptional changes in response to heat shock. Note that the y-axis scale varies across panels to improve visualization of the differences in gene expression dynamics between cell lines and batches.

Histograms (Figure 3) illustrate significant differences in DEG counts, with HepG2 cells consistently showing higher numbers of DEGs than HEK293 and HeLa cells. For batch 1 (Figure 3 top panels), the DEG counts for HEK293 were 3444 (0 h vs. control), 2096 (8 h vs. control) and 3802 (8 h vs. 0 h). Similarly, HeLa showed 2168, 1590 and 3916 DEGs for these comparisons, respectively, while HepG2 exhibited 4609, 9948 and 9219 DEGs. In batch 2 (Figure 3 bottom panels), HEK293 had 1639, 947 and 2955 DEGs; HeLa had 2613, 4877 and 5076 DEGs; and HepG2 exhibited 3547, 9665 and 8791 DEGs for the exact comparisons.

**Figure 4:**
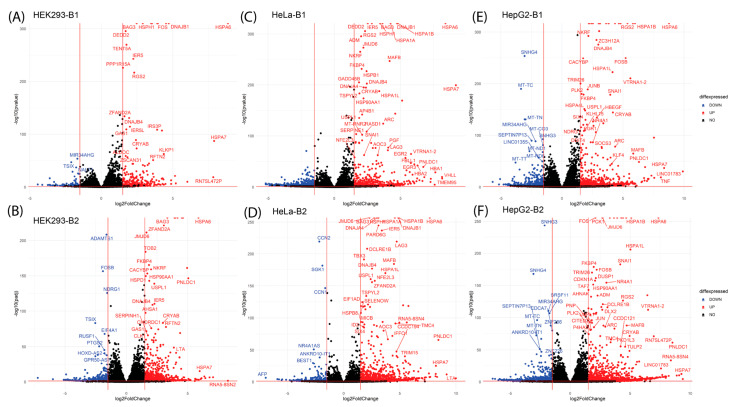
Volcano plots show the distribution of DEGs for condition comparisons within each cell line. Each point represents a gene, with the x-axis indicating log2 fold change (magnitude of expression change) and the y-axis representing statistical significance (negative log10 adjusted *p*-value). Genes upregulated (log2FC > 1) are shown in red, while downregulated genes (log2FC < −1) are in blue. Examples include HEK293 (**A**,**B**), HeLa (**C**,**D**), and HepG2 (**E**,**F**) comparisons at 0 h post-heat shock vs. control, visualizing the most significant transcriptional changes.

Volcano plots (Figure 4 and Supplementary Figure S2) visualized DEGs’ fold change and statistical significance across comparisons. This approach highlights the fold change and statistical significance of gene expression for each comparison: in HEK293, 1867 genes were overexpressed, and 1577 genes were underexpressed in the 0hrvsCnt condition for Batch 1. In HeLa, 1661 genes were overexpressed, and 507 were underexpressed in the 0 h vs. Cnt condition for Batch 1. In HepG2, 2188 genes were overexpressed, and 2421 genes were underexpressed in the 0 h vs. Cnt condition for Batch 1.

Key heat shock response genes, including HSPA6, HSPA1A, HSPA1B, BAG3, and DNAJB1, were significantly overexpressed across all cell lines and conditions, with HepG2 cells demonstrating the most robust transcriptional response. These genes play critical roles in protein folding, chaperoning, and cellular recovery from stress, highlighting their centrality in the heat shock response. Notably, HSPA6 and HSPA1A exhibited a continuous increase in expression at 8 h post-recovery in HepG2 cells, contrasting with the other cell lines, where their expression dropped at 8 h (remaining higher than control but lower than 0 h). This distinct pattern suggests a prolonged activation of the heat shock response in HepG2 cells, potentially reflecting their reliance on enhanced proteostasis under stress. A comprehensive summary of HSP expression, including log2 fold changes and adjusted *p*-values, is provided in Supplementary Table S1.

**Figure 5:**
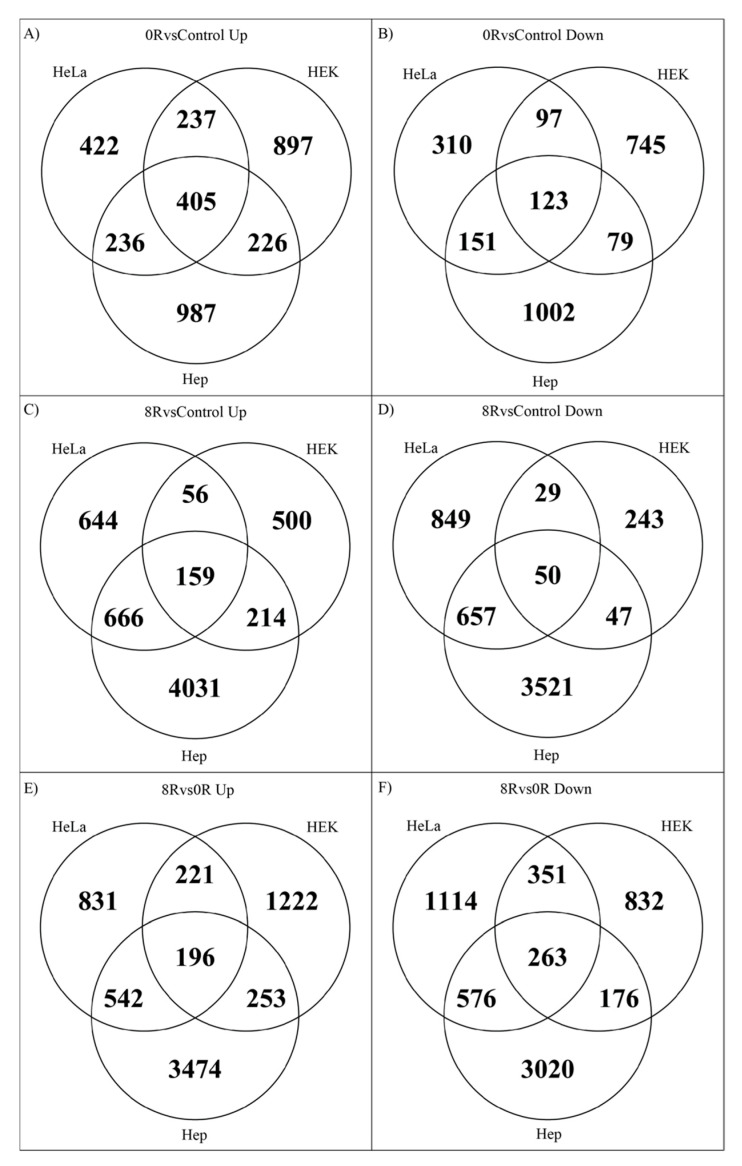
Venn diagrams summarizing conserved DEGs between cell lines under specific conditions. The size of overlapping circles corresponds to the number of shared genes across comparisons. Overexpressed genes (log2FC > 0.5; *p*-adj. < 0.05) and underexpressed genes (log2FC < −0.5; *p*-adj. < 0.05) are visualized for 0 h post-heat shock vs. control (**A**,**B**), 8 h vs. 0 h (**C**,**D**), and 8 h vs. control (**E**,**F**).

The next step involved identifying conserved genes shared between the cell lines to further elucidate the similarities in gene expression during mild heat shock across HeLa, HEK, and Hep cells. Conserved DEGs across all three cell lines and batches included 405 genes overexpressed at 0 h post-heat shock vs. Control, 196 genes overexpressed at 8 h post-heat shock vs. 0 h, and 159 genes overexpressed at 8 h post-heat shock vs. Control (Figure 5A,C,E).

Underexpressed genes shared across all cell lines and batches included 123 genes at 0 h post-heat shock vs. Control, 263 genes at 8 h post-heat shock vs. 0 h, and 50 genes at 8 h post-heat shock vs. Control (Figure 5B,D,F).

### 2.3. Functional Enrichment Analysis

**Figure 6:**
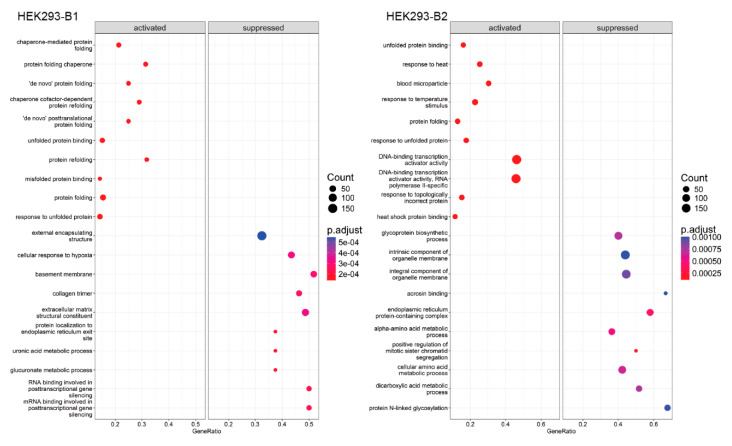
Dot plot visualization of Gene Set Enrichment Analysis (GSEA) results**.** The top 15 positively and negatively enriched gene sets, ranked by normalized enrichment scores (NES), are shown for HEK293 at 0 h post-heat shock vs. control (Batch 1 and Batch 2). Each dot represents a gene set, with size proportional to the number of enriched genes and color reflecting the statistical significance (adjusted *p*-value).

To elucidate biological processes associated with DEGs, we performed functional enrichment analyses using GSEA and STRING, conducting these analyses separately for each batch due to batch effects. Dot plots (Figure 6 and Supplementary Figure S3) highlight the top ten gene sets with the highest and lowest normalized enrichment scores (NESs) across comparisons. In HEK293 (0 h vs. Control), enriched gene sets related to protein folding and response to unfolded proteins were consistently observed in both batches. However, HeLa and HepG2 showed less overlap in enriched gene sets between batches, suggesting a more significant batch-dependent variability.

**Figure 7:**
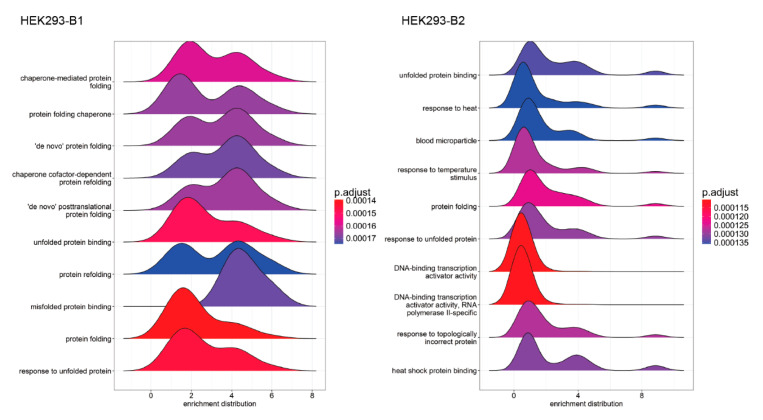
Distribution of Log2 Fold Changes in Enriched Gene Sets. Distribution of log2 fold changes for the top 15 positively enriched GSEA hits in HEK293, HeLa, and HepG2 cell lines at 0 h post-heat shock vs. control. The peak height represents the number of genes within each range of log2 fold change, highlighting the degree of enrichment within gene sets. Separate plots depict Batch 1 and 2 results for each cell line.

Distribution plots (Figure 7 and Supplementary Figure S4) of enriched genes within the top 15 NES gene sets revealed similar enrichment profiles for genes involved in protein folding and chaperone activity in HEK293. In contrast, unique enrichment distributions were observed in HeLa and HepG2, reflecting differences in stress response pathways.

**Figure 8:**
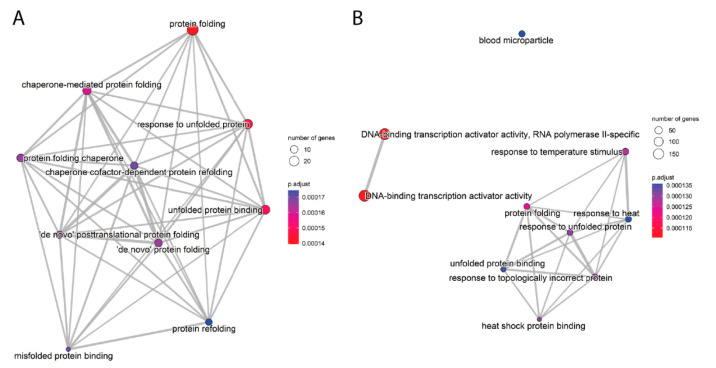
Enrichment maps of the top 10 positively enriched gene sets (GSEA NES) for HEK293 cells at 0 h post-heat shock vs. control. Each node represents a gene set, with edges indicating shared genes between sets. The network visualizes functional overlap and relationships among the most significant pathways for each batch. (**A**) protein folding and (**B**) protein folding and DNA-binding transcription activator activity).

Enrichment maps (Figure 8 and Supplementary Figure S5C–F) visualized interactions among enriched gene sets. In HEK293, gene sets related to protein folding and heat shock response formed cohesive clusters. In HeLa and HepG2, fewer interconnections were observed, with unexpected enrichments in immune-related pathways, such as B cell-mediated immunity and immunoglobulin receptor binding, in HepG2.

A combined analysis of enriched Gene Ontology (GO) terms across HEK293, HeLa, and HepG2 cells revealed conserved and cell-line-specific responses to heat shock (Supplementary Figures S6 and S7). Universal enrichment in “response to unfolded protein”, “protein folding”, and “response to heat” highlights shared proteostasis mechanisms critical for managing proteotoxic stress. HEK293 cells uniquely emphasized transcriptional regulation and proteome stability, with enrichment in “RNA catabolic process” and “negative regulation of transcription from RNA polymerase II promoter”. HeLa cells included proteostasis and metabolic flexibility, with pathways like “response to topologically incorrect protein” and “response to oxygen levels”, reflecting tumor-specific adaptations. HepG2 cells displayed liver-specific adaptations, including “response to glucocorticoid” and “fat cell differentiation”, alongside general stress responses, demonstrating a dual focus on proteotoxicity and metabolic regulation.

Although several pathways were unique, we also identified some conserved gene sets enriched across all cell lines and conditions. Compared to the control sample, four gene sets were enriched and shared between HEK293, HepG2, and HeLa cell lines immediately following mild heat shock. These gene sets are Receptor Ligand Activity (GO:0048018), Signaling Receptor Activator Activity (GO:0030545), Protein Folding Chaperone (GO:0044183), and Class A/1 Rhodopsin-like Receptors (HSA-373076) (Table 1 and Supplementary Table S2. Notably, the Receptor Ligand Activity pathway (GO:0048018) was consistently enriched across all conditions, cell lines, and batches (Table 1).

### 2.4. Analysis of Receptor Ligand Activity (GO:0048018) Genes

**Figure 9:**
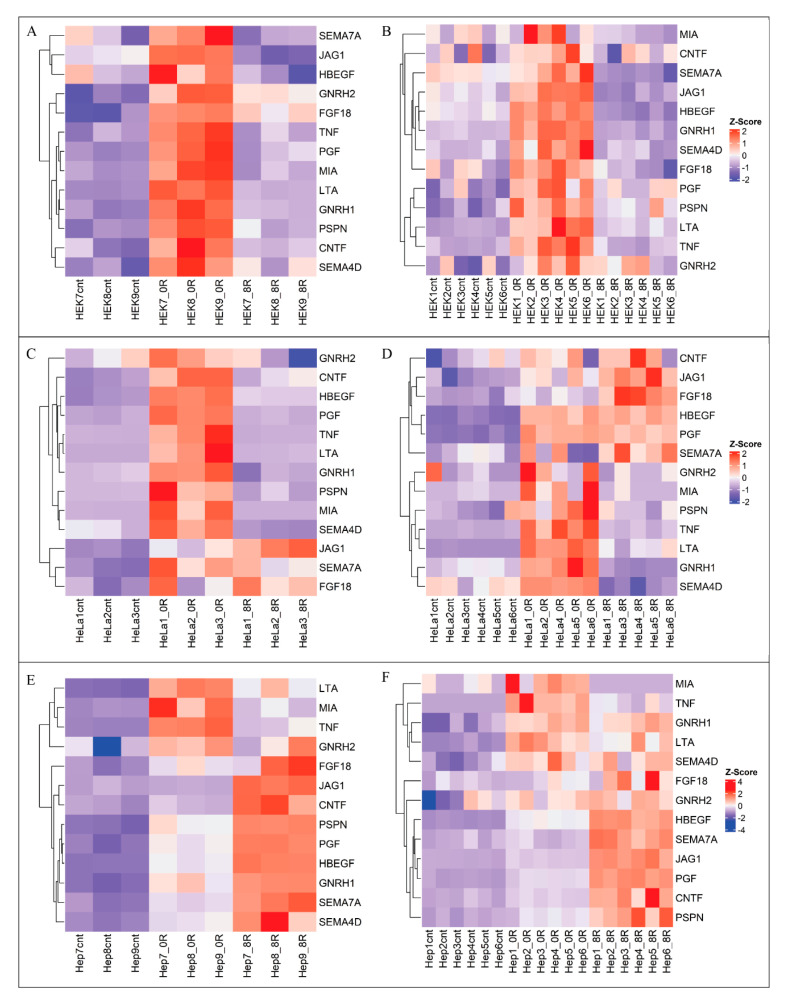
Distribution of Log2 Fold Changes in Enriched Gene Sets. Heatmaps of Z-score-scaled log2 fold changes for 13 conserved genes within the Signal Receptor Ligand Activity (GO:0048018) pathway. Rows represent genes, while columns correspond to cell lines and conditions. Colors correspond to Z-scores, with red indicating positive Z-scores (maximal upregulation) and blue indicating negative Z-scores (maximal downregulation). Separate heatmaps are provided for Batch 1 (**A**,**C**,**E**) and Batch 2 (**B**,**D**,**F**), illustrating consistent patterns of gene regulation across batches and cell lines.

**Figure 10:**
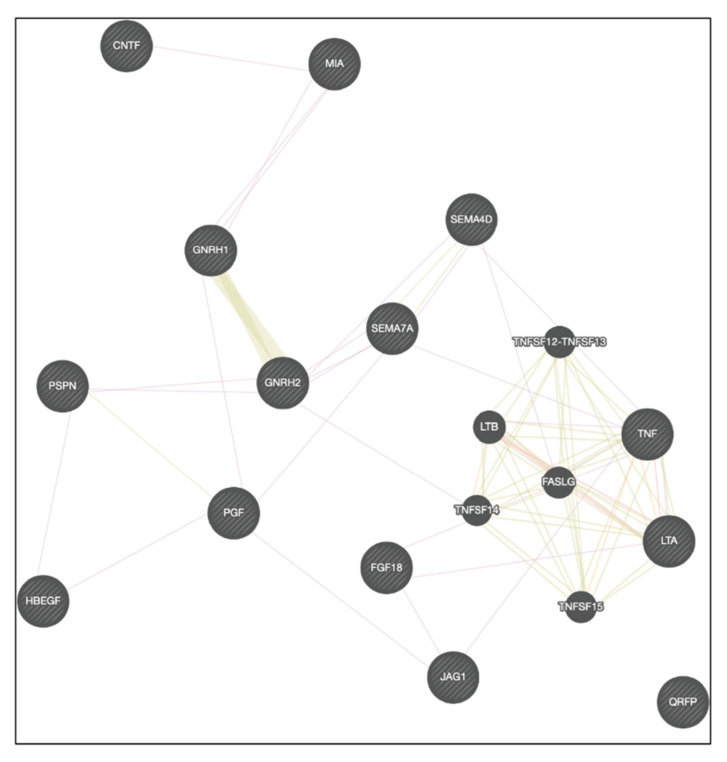
Network Map of GO:0048018 Pathway Genes. Cytoscape-generated network map of the 13 conserved DEGs within the Signal Receptor Ligand Activity (GO:0048018) pathway. Nodes represent genes, and edges indicate predicted functional interactions, offering a systems-level view of pathway dynamics.

Of the 75 genes associated with the Receptor Ligand Activity pathway, 13 were consistently expressed across both batches and all three cell lines under each condition. These genes, including *TNF*, *GNRH2*, *PSPN*, *MIA*, *SEMA4D*, and *HBEGF*, are involved in cell survival, repair, and growth processes (Figure 9). Interaction networks (Figure 10) further illustrate how these genes interact to coordinate stress responses. As anticipated, the gene expression patterns of the conserved genes are similar between the cell lines and batches throughout heat shock. However, some variations in the magnitude of expression can be observed. Supplementary Table S3 provides an overview of the biological processes associated with these 13 genes, many promoting cell survival, repair, and growth.

### 2.5. Gene Expression Assessment via qPCR

We conducted qPCR on selected genes to validate the DGE analysis findings. This approach confirmed our transcriptomic results and provided additional insight into the induction of the heat shock response. The *Heat Acclimation* gene set (GO:0010286) was chosen for its relevance, containing six genes known to exhibit significant expression changes during heat shock.

**Figure 11:**
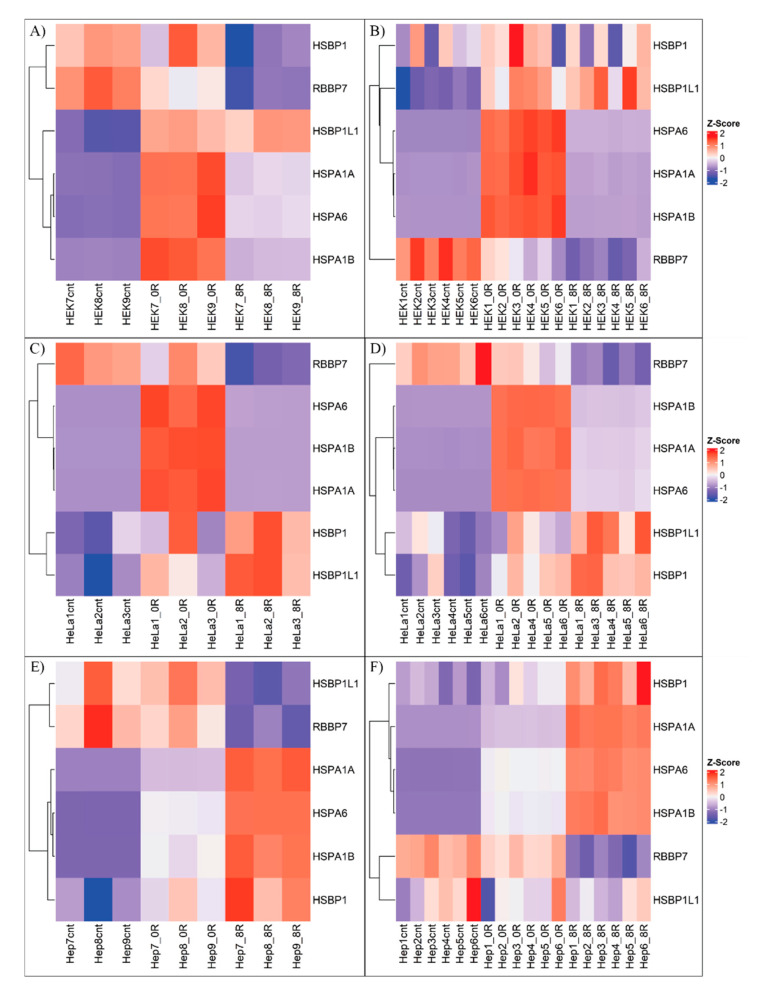
Heatmaps of Heat Acclimation Genes Across Conditions**.** Heat maps show expression changes for Heat Acclimation (GO:0010286) genes at control, 0 h post-heat shock, and 8 h post-heat shock conditions. Colors correspond to Z-scores, with red indicating positive Z-scores (maximal upregulation) and blue indicating negative Z-scores (maximal downregulation). Separate heatmaps are provided for Batch 1 (**A**,**C**,**E**) and Batch 2 (**B**,**D**,**F**) across HEK293, HeLa, and HepG2 cell lines.

Figure 11 summarizes the expression changes in HEK293 (Figure 11A,B), HeLa (Figure 11C,D), and HepG2 (Figure 11E,F) cell lines across batches and conditions. These results strongly support the activation of the heat shock response. *HSPA1A, HSPA1B,* and *HSPA6* were consistently upregulated across all cell lines, while *RBBP7* was downregulated. However, expression patterns of *HSBP1* and *HSBP1L1* varied. In HEK293 cells, *HSBP1* decreased at 0- and 8-h post-heat shock, whereas it increased in HeLa and HepG2 cells under the same conditions. Conversely, *HSBP1L1* increased in HEK and HeLa cells but exhibited batch-specific behavior in HepG2, decreasing in Batch 1 at 8 h and remaining unchanged in Batch 2. Despite this variability, the consistent expression trends of *HSPA1A, HSPA1B, HSPA6,* and *RBBP7* across all conditions underscore the canonical activation of the heat shock response.

qPCR analysis was further performed on mRNA from HeLa cells at 0- and 8-h post-heat shock, along with control samples maintained at 37 °C. The primers targeted four “Heat Acclimation” genes (*HSPA1A*, *HSPA6*, *BAG3*, *DNAJB1*) and three “Signal Receptor Ligand Activity” genes (*LTA*, *MIA*, *TNF*), with *ACTB* and *GAPDH* as reference controls.

**Figure 12:**
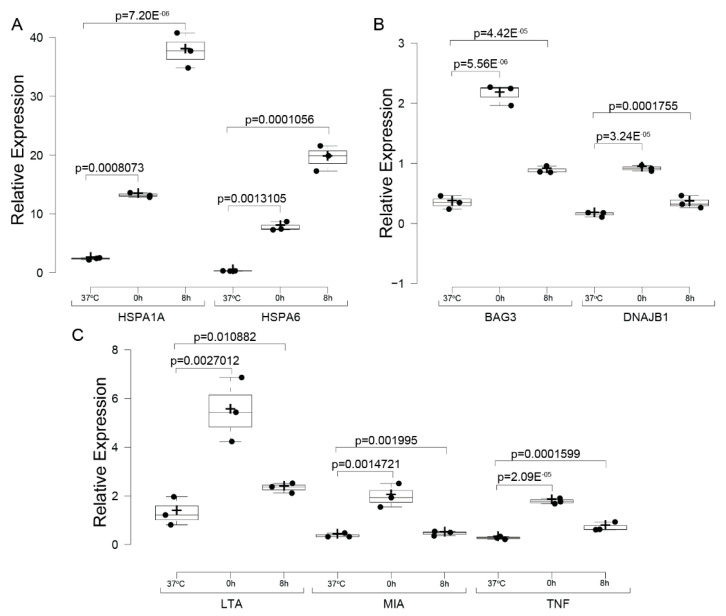
qPCR Validation of Key Gene Sets. mRNA fold change measured by qPCR for Heat Acclimation (GO:0010286) and Signal Receptor Ligand Activity (GO:0048018) genes in HeLa cells at control, 0 h post-heat shock, and 8 h post-heat shock. Results are normalized against housekeeping genes (ACTB and GAPDH). Heat shock genes (HSPA1A, HSPA6) exhibit the highest induction, while signal receptor ligand genes (LTA, MIA, TNF) show transient upregulation (**A**–**C**). Note that the Y-axis scale varies across panels. The experiment was repeated using three biological replicates (black dots). Statistical significance was determined using one-way ANOVA (Analysis of Variance) followed by post-hoc Tukey HSD (Honestly Significant Difference) and Bonferroni tests.

As shown in Figure 12, *HSPA1A* and *HSPA6* exhibited the highest fold changes at 0- and 8 h post-heat shock (Figure 12A), aligning with their central roles in the heat shock response. *BAG3* and *DNAJB1* also displayed significant changes in expression (Figure 12B). For the “Signal Receptor Ligand Activity” genes, *LTA, MIA,* and *TNF* were upregulated immediately after heat shock but returned to near-control levels by 8 h post-recovery (Figure 12C). The observed patterns closely mirrored those revealed by RNA-seq analyses, except HSPA1A and HSPA6, which continued to increase at 8 h post-recovery in the qPCR data. Although the log2 fold change values varied slightly between the two methods, the overall trends were consistent (Supplementary Figure S8). Differences between RNA-seq and qPCR results, particularly for HSPA1A and HSPA6 at 8 h post-recovery, highlight the complementary nature of these methods. RNA-seq provides a transcriptome-wide perspective, normalized across all detected genes, while qPCR focuses on absolute expression changes relative to housekeeping genes, offering enhanced sensitivity for specific transcripts. The observed differences suggest that while RNA-seq indicates a relative decrease from the immediate heat shock response at 0 h, qPCR reflects the ongoing transcriptional activity of these genes at 8 h, underscoring the dynamic nature of their regulation during recovery.

These results validate the involvement of key “Heat Acclimation” and “Signal Receptor Ligand Activity” genes in the heat shock response. The consistency between qPCR findings and DGE and functional enrichment analyses reinforces the robustness of our conclusions and highlights specific pathways central to stress adaptation mechanisms.

## 3. Discussion

This study characterizes the heat shock response (HSR) in three human cell lines—HeLa, HEK293, and HepG2—by subjecting them to mild heat shock and examining recovery periods of 0 and 8 h. Using principal component analysis (PCA), differential gene expression (DGE), and functional enrichment analyses, we identified conserved and cell-specific genes and pathways activated during and after heat shock. PCA revealed that the primary variance in gene expression was driven by cell type, batch effects, and heat shock recovery time points (Figure 1 and Figure 2). To address the loss of statistical power caused by splitting into two experimental batches, we analyzed the batches individually, effectively gaining a second independent repetition and ensuring that observed trends were consistent across both sets of experiments.

These findings align with prior transcriptomic studies, which emphasize cell type [21,22] and experimental conditions [21,23] as dominant sources of variance in gene expression under stress. Notably, our results highlight how heat shock as a specific stressor influences transcriptomic variability, echoing previous research that identified stressor-specific transcriptional signatures while reaffirming the universal role of cell type as a major determinant of gene expression patterns [13,21,24,25].

Variability in DEG counts across batches and conditions reflects the dynamic nature of the heat shock response (HSR) and the inherent biological and technical factors influencing transcriptomic analyses [26,27]. Differences in sample size and experimental timing between batches likely contributed to these discrepancies [13,28]. Despite these discrepancies, consistent trends across batches reinforce the robustness of our findings and the conserved nature of key HSR pathways.

While the heat shock response was conserved across all cell lines, transcriptional responses suggested cell-type-specific adaptations. Universal enrichment in proteostasis pathways indicates shared strategies for maintaining protein integrity under heat stress, while cell-specific differences point to unique biological priorities. In HEK293 cells, the data suggest a focus on transcriptomic and proteomic stability to support homeostasis, whereas HeLa cells appear to utilize proteostasis and metabolic reprogramming to sustain rapid proliferation. HepG2 cells show evidence of liver-specific adaptations, including glucocorticoid signaling and differentiation processes, highlighting a dual focus on systemic regulation and localized stress responses. These differences may be reflective of the inherent stress environments that cancer cells encounter, such as hypoxia [29] and metabolic dysregulation [29,30]. Cancer cells like HeLa and HepG2 are known to rely on enhanced proteostasis to maintain homeostasis under stress [31], which may explain their more dynamic transcriptional response compared to non-cancerous HEK293 cells [31,32,33,34,35,36,37].

A consistent finding was the enrichment of the “Receptor Ligand Activity” pathway across all conditions and cell lines. Traditionally associated with extracellular signaling, this pathway may mediate cellular responses to proteotoxic stress by coordinating adaptive responses such as apoptotic regulation and survival signaling [38,39]. For example, genes like TNF and HBEGF contribute to inflammatory signaling and tissue repair, while PSPN and MIA support survival under adverse conditions [40,41,42,43]. In multicellular systems, receptor-ligand interactions may synchronize stress responses across cell populations, balancing survival, and apoptosis to maintain tissue integrity [44,45,46]. Further exploration of receptor-ligand pairs involved in the HSR could uncover novel mechanisms of stress adaptation and resilience, offering potential therapeutic strategies targeting stress communication networks. Identifying receptors uniquely or commonly expressed on the surfaces of HeLa, HepG2, and HEK293 cells could provide valuable insights into how receptor-ligand signaling mediates the HSR. Receptors conserved across all cell lines or specific to cancer cells could represent likely candidates for immediate regulation by the HSR, either following heat shock or during recovery. Targeting receptor-ligand signaling could offer a novel therapeutic strategy by disrupting stress communication networks, complementing existing therapies such as heat shock protein inhibitors to reduce tumor resilience to proteotoxic stress.

The upregulation of heat shock protein (HSP) genes, including HSPA1A, HSPA1B, and HSPA6, underscores their central role in managing proteotoxic stress across all cell lines. These genes support proteome stability in HEK293 cells but are more dynamically expressed in HeLa and HepG2 cells, reflecting their dependence on HSPs to withstand chronic stress conditions. This reliance on HSPs highlights their potential as therapeutic targets, particularly in cancer cells, where stress response pathways are critical for resilience.

It is important to note that these experiments were conducted in established cell lines, which, while offering precise control over experimental variables, lack the complexity and tissue-level interactions present in primary cells or animal models. This limitation restricts our ability to fully capture the influence of microenvironmental factors, cell-cell interactions, and in vivo stress responses. Future studies incorporating primary cells or in vivo models could provide deeper insights into how these pathways function in more physiologically relevant settings [47].

In conclusion, this study highlights the conserved nature of cellular stress responses across diverse cell types and the unique adaptations cancer cells employ to survive under stress. The consistent activation of “Receptor Ligand Activity” offers a novel perspective on stress communication mechanisms and their role in cancer resilience. By uncovering both conserved pathways and cell-type-specific adaptations, this study lays the groundwork for developing interventions that exploit the vulnerabilities of stress-adapted cells.

## 4. Materials and Methods

### 4.1. Cell Culture

To examine the differential heat shock response, we utilized three human cell lines: human embryonic kidney cells (HEK293; ATCC^®^ CRL-1573™) (American Type Culture Collection, Manassas, VA, USA), HeLa cells derived from Henrietta Lacks (ATCC^®^ CCL-2™), and hepatocellular carcinoma cells (HepG2; ATCC^®^ HB-8065) obtained from ATCC in December 2016 and verified bi-annually. HEK293 cells were cultured in Dulbecco’s Modified Eagle Medium (DMEM; Corning, Corning, NY, USA), while HeLa and HepG2 cells were grown in Minimum Essential Medium (MEM; Corning, Corning, NY, USA). Media for both cell lines were supplemented with 10% fetal bovine serum (FBS; Gibco, Thermo Fisher Scientific, Waltham, MA, USA), 2 mM L-glutamine, and penicillin-streptomycin. HeLa and HepG2 media were supplemented with 0.1 mM non-essential amino acids (NEAA; Corning, Corning, NY, USA) and 1 mM sodium pyruvate (Corning, Corning, NY, USA). All cultures were maintained in a humidified atmosphere containing 5% CO_2_ at 37 °C.

### 4.2. Heat Shock Treatment

To assess the effect of heat shock on transcription, cells were either maintained at 37 °C or subjected to heat stress at 42 °C for 60 min in a humidified CO_2_ incubator. Following heat shock, cells were allowed to recover at 37 °C for 8 h. This procedure was independently conducted on HeLa, HEK293, and HepG2 cell lines. Newly thawed, low-passage cells (passages 4–7) were cultured in 175 cm² flasks until reaching approximately 80% confluency. For each experimental condition, three flasks were used: one remained at 37 °C as a control, while two others were exposed to 42 °C for one hour to induce heat shock. Of the heat-shocked flasks, one was processed immediately after heat shock (0 h recovery, 0R), and the other was allowed to recover at 37 °C for 8 h (8 h recovery, 8R) (Supplementary Figure S9). This procedure was repeated using freshly thawed cells to generate biological replicates. After treatment, cells were harvested through trypsinization, pelleted, and frozen for subsequent analysis.

### 4.3. Sample Preparation, cDNA Library Preparation, and Sequencing

Cells from the control, 0R, and 8R conditions were sent to Novogene (Sacramento, CA, USA) for RNA isolation, library preparation, and sequencing. Total RNA was isolated using the Qiagen RNeasy series kit (Hilden, Germany), and quality control (QC) was meticulously performed at each step to ensure reliable data. RNA degradation and contamination were assessed on 1% agarose gels, purity was verified using a NanoPhotometer^®^ spectrophotometer (IMPLEN, Westlake Village, CA, USA), and integrity and quantification were evaluated using the RNA Nano 6000 Assay Kit on the Bioanalyzer 2100 system (Agilent Technologies, Santa Clara, CA, USA). For transcriptome sequencing, 1 μg of total RNA per sample was used to generate sequencing libraries with the NEBNext^®^ UltraTM RNA Library Prep Kit for Illumina^®^ (NEB, Ipswich, MA, USA) following the manufacturer’s protocol. mRNA was isolated using poly-T oligo-attached magnetic beads, fragmented under elevated temperature, and reverse transcribed into cDNA. First and second-strand cDNA synthesis involved random hexamer primers, M-MuLV Reverse Transcriptase, DNA Polymerase I, and RNase H, followed by end repair, adenylation, and ligation of NEBNext adaptors. cDNA fragments of 150–200 bp were selected using AMPure XP beads (Beckman Coulter, Brea, CA, USA), processed with USER Enzyme, and amplified via PCR with Phusion High-Fidelity DNA polymerase and indexed primers. Libraries were purified with the AMPure XP system and validated on the Agilent Bioanalyzer 2100 system. Clustering was conducted on a cBot Cluster Generation System using a PE Cluster Kit cBot-HS (Illumina, San Diego, CA, USA), and paired-end sequencing was performed on an Illumina NovaSeq S4 PE100 platform, generating at least 30 million reads per sample across all conditions.

### 4.4. Transcriptomics Analyses (Detailed Methodology in Supplementary Data S1)

Two experimental batches were conducted to evaluate the heat shock response (HSR) in three cell lines (HEK293, HeLa, and HepG2). Batch 1 included three biological replicates per condition, and Batch 2 included six biological replicates per condition. Power analysis using powsimR showed that three and six replicates explain ~58% and ~70% of DEGs, respectively, at an FDR of 15% and 10%. While combining the batches would theoretically provide greater statistical power (~75% of DEGs at 8% FDR), the experiments were conducted separately, leading to batch-specific variability. To address this, batches were analyzed independently, ensuring trends and biological patterns observed in the datasets were consistent and reproducible.

**Read Quality Control Overview:** Adapter sequences, PhiX library sequences, and low-quality reads are common byproducts of library preparation and next-generation sequencing (NGS). To ensure that high-quality reads are used for downstream analysis, BBDuk was employed to perform adapter trimming, PhiX filtering, and quality filtering based on Phred scores [48,49].

**Adapter and PhiX Trimming:** To eliminate unwanted sequences from downstream analysis, we used BBDuk’s k-mer trimming functionality for both adapter and PhiX filtering. Each paired read was processed with a BBDuk shell script to remove reads that matched reference k-mers associated with adapter sequences or the PhiX control library.

**Phred Quality Score:** To ensure high-quality reads were retained, BBDuk’s Phred quality trimming function was applied. This function scans the right end of paired reads, trimming until the Phred quality score meets the specified threshold. If the score remains unsatisfactory, the entire paired read is discarded. These processes were executed via command line for each pair of reads.

**Reference Genome Indexing and Alignment of Reads to an Indexed Reference Genome:** To quantify gene expression, it is essential to map high-quality reads to the human reference genome to identify the features in the dataset. STAR was employed for this purpose, accepting reads cleaned by BBDuk and outputting binary sequence alignment map files (.BAM) after performing splice-aware mapping against the indexed human reference genome (GRCh38.p13) [50]. Before alignment, the indexed reference genome was generated.

**Sort and Index Mapped Reads for Feature Counting:** Samtools was used to sort an input .BAM file and create a corresponding index file (.BAI), which is necessary for downstream feature counting steps [51]. The splice-aware and mapped .BAM files generated by STAR, as detailed in “Reference Genome Indexing and Alignment of Reads to an Indexed Reference Genome”, served as the input.

**Feature Counts:** HTSeq was utilized to create a raw gene count matrix by determining the number of reads mapped to each feature. The inputs required for HTSeq include a sorted aligned .BAM file, its corresponding index .BAI file, and a reference human genome annotation file (GRCh38.104.gtf) [52]. The sorted and indexed alignment files were produced as outlined in “Sort and Index Mapped Reads for Feature Counting”.

**Quality Control:** BBDuk, STAR, Samtools, and HTSeq generated multiple output files distributed across various directories. To summarize and evaluate these files, MultiQC was utilized, which provides an overview of file types associated with next-generation sequencing read processing [53]. By running MultiQC from a directory containing all relevant subdirectories with output files, users can verify the expected number of files and their types for each sample. Supplementary Tables S4 and S5 display the quality control metrics for each sample in batch 1 and batch 2, respectively.

**Generating and Visualizing DEGs:** The negative binomial distribution is a suitable model for analyzing raw count data that is well-dispersed and influenced by multiple sources of variance, making DESeq2 an ideal choice for identifying differentially expressed genes (DEGs) [54]. DESeq2 estimates the mean and models the dispersion of gene expression for each gene observed in the dataset.

**Gene Counts Normalization and Dimensionality Reduction:** Utilizing DESeq2 for differential gene expression (DGE) analysis allows researchers to leverage its built-in variance stabilizing transformation (VST), which helps reduce the impact of outliers and addresses heteroscedasticity [54]. The VST-normalized counts were subsequently used as inputs for dimensionality reduction analyses. Principal component analysis (PCA) and row-scaled heatmap analysis were employed as dimensionality reduction techniques. These methods were chosen to effectively visualize the intricate VST-normalized gene expression data, encompassing over 20,000 gene features across nine samples, into a more concise and interpretable format. PCA was conducted to identify the primary sources of variation within the dataset and to estimate the effect sizes between samples based on the gene features contributing to that variance. PCA was selected as the dimensionality reduction technique for this study due to its ability to effectively summarize high-dimensional data while retaining the principal variance in gene expression patterns. This technique is computationally efficient and widely used in transcriptomic studies to identify clustering patterns and primary drivers of variance. To ensure robustness, we evaluated PCA results across multiple normalization methods, including Variance Stabilizing Transformation (VST), log-transformation, and Counts Per Million (CPM). The clustering patterns and primary drivers of variance remained consistent across all normalization methods, confirming the reliability of the observed results. PCA was conducted using R’s prcomp function, along with the tidyverse and ggfortify libraries.

**Histograms, Volcano Plots, and Venn Diagrams:** DEGs were acquired from the normalized log2FC data created by DESeq2. Separating by cell line, the over and under-expressed genes for each condition comparison (0R vs. Control, 8R vs. Control, 8R vs. 0R) were categorized as log2FC > 0.5 and log2FC < 0.5, respectively. These were plotted on a histogram in R using ggplot2 to visualize the number of DEGs present within a cell line at each pairwise condition comparison. Volcano plots were then generated for each condition pairwise comparison with R’s ggplot2 library to visualize statistically significant fold changes in gene expression. To visualize the significant batch conserved DEGs within each cell line at a given condition, Venn diagrams were created using tidy verse’s ggplot2 library. Significant DEGs were defined as having a |log2FC| > 0.5 and a *p*. adj. < 0.05 (Benjamini-Hochberg). Tables with DEGs from all comparisons are in Supplementary Data S2.

**Functional Enrichment Analysis Using GSEA:** Gene Set Enrichment Analysis (GSEA) was run against the ontological gene set collections (C5) defined by the molecular signatures database [55,56]. GSEA inputs ranked log2FC outputs from DESeq2 and designated an enrichment score for each pathway within the gene set collection. Enrichment scores are defined by increases to a running-sum statistic when a gene is in the gene set and decreases when it is not; the normalized enrichment scores (NESs) then enable comparison across gene sets by accounting for differences in set size and correlations between the gene set and expression dataset. For this study, only human collections were used. For this reason, the normalized enrichment score was used in this analysis. Tables with GSEA analyses are in Supplementary Data S2.

**Functional Enrichment Analysis using STRING:** STRING analysis investigated protein–protein interactions across various batches, cell lines, and condition comparisons. The STRING database aggregates, scores, and integrates publicly available information on protein–protein interactions, leveraging these data sources to generate predictive models of interaction networks [57]. We used our differentially expressed gene (DEG) data obtained from DESeq2 to carry out an enrichment analysis. To determine conserved pathways, the tables were compared, and genesets present in both batches and all three cell lines at a given condition were compiled in a table. Tables with STRING analyses are in Supplementary Data S2.

**Visualization of Gene Expression Patterns Using Heatmaps:** Row scaled heatmaps built with the Complexheatmap library were used to visualize the VST normalized expression patterns of genes found in “Heat Acclimation” and “Receptor Ligand Activity” gene sets. Thirteen genes from “Receptor Ligand Activity” with conserved gene expression in both batches, three cell lines, and three condition pairwise comparisons were identified. All six genes from “Heat Acclimation” were visualized with row scaled heatmaps for each batch [54,58].

**Cytoscape Generated Network:** Cytoscape’s [59] predicted network analysis was performed to determine the interactions of genes based on the acquired expression data. The network analysis uses a list of DEGs and the known interaction networks in the MSigdbr database.

### 4.5. Molecular Validation of Using qPCR

Following the manufacturer’s protocol, RNA was isolated using 4 million HeLa cells per condition (control cells, 0 h recovery, 8 h recovery; different batches from the ones used for RNA-seq) using the Direct-Zol RNA mini-prep Kit (ZymoResearch, Irvine, CA, USA). Following the manufacturer’s protocol, cDNA was synthesized from 1 ug of total RNA using the Superscript IV First-Strand synthesis system (ThermoFisher Scientific, Waltham, MA, USA) and Oligo (dT)_20_ primers. cDNA samples were diluted to a concentration of 50 ng/uL. qPCR reactions were prepared with the Power SYBR™ Green PCR Master Mix (ThermoFisher Scientific, Waltham, MA, USA) according to the manufacturer’s instructions. Three biological replicates were run for each gene and condition [Gene names and primers (generated using NCBI’s primer-blast utility) are shown in Supplementary Table S6]. qPCR was performed using the CFX96 Touch Real-Time Detection System (Bio-Rad, Hercules, CA, USA). The relative normalized expression [60] of the raw transcript levels was calculated using the Livak method for each gene [61] using the software provided with the instrument [62]. The reference genes used in this method were ACTB and GAPDH. Statistical significance was assessed using one-way ANOVA (Analysis of Variance) followed by post-hoc Tukey HSD (Honestly Significant Difference) and Bonferroni tests. A *p* value < 0.05 was considered statistically significant. Results were plotted via boxplot using BoxPlotR [63].

## Figures and Tables

**Table 1:** Enriched gene sets were conserved in both batches and HEK293, HepG2, and HeLa cell lines (associated functions can be found in Supplementary Table S2).

0R vs. Control		8R vs. Cnt		8R vs. 0R	
ID	Name	ID	Name	ID	Name
GO:0048018	Receptor Ligand Activity	GO:0048018	Receptor Ligand Activity	GO:0048018	Receptor Ligand Activity
GO:0030545	Signaling receptor activator activity	hsa04080	Neuroactive ligand-receptor interaction	hsa04080	Neuroactive ligand-receptor interaction
GO:0044183	Protein folding chaperone	HSA-500792	GPCR ligand binding	HSA-500792	GPCR ligand binding
HSA-373076	Class A/1 (Rhodopsin-like receptors)			HSA-373076	Class A/1 (Rhodopsin-like receptors)

## Data Availability

All data reported are provided in the text, Supplementary Figures, or Supplementary Data S1 and S2. The raw data are hosted at NCBI (GEO: GSE285497). The scripts used can be found at (https://github.com/ajreinschmidt/MSc-Code/blob/main/iSEE%20Analysis%20Script.R, accessed on 1 January 2024).

## Supplementary Materials

The following supporting information can be downloaded at: https://www.mdpi.com/article/10.3390/ijms26031057/s1.
