# Supplementary material for: Transcriptomics Unveil Canonical and Non-Canonical Heat Shock-Induced Pathways in Human Cell Lines
Source: Int J Mol Sci. 2025 Jan 26;26(3):1057. doi: 10.3390/ijms26031057 (PMC11816735; doi:10.3390/ijms26031057)
Supplement: Supplementary file 1 [file ijms-26-01057-s001.zip › Supplemental Materials-R1.pdf]

Supplemental Materials

Title

Transcriptomics Unveil Canonical and Non-Canonical Heat Shock-Induced Pathways in Human Cell Lines

Authors

Andrew Reinschmidt[1], Luis Solano[1†], Yonny Chavez[1], William Drew Hulsy[1], Nikolas Nikolaidis[1*]

Affiliations

[1] Department of Biological Science, Center for Applied Biotechnology Studies, and Center for Computational and Applied Mathematics, California State University Fullerton, Fullerton, CA, USA.

Andrew Reinschmidt, areinschmidt4@csu.fullerton.edu
Yonny Chavez, Yonny@csu.fullerton.edu
William Drew Hulsy, wdhulsy@csu.fullerton.edu

[†] Current address: Center for Complex Biological Systems, University of California, Irvine
Luis Solano, lesolano@uci.edu

[*] Corresponding author, Nikolas Nikolaidis, nnikolaidis@fullerton.edu

Supplementary Table 1. Expression of the major heat shock protein genes in HEK293, HeLa, and HepG2 after heat shock.

Please see separate excel file: Supplementary Table 1.

Supplementary Table 2. The function of the conserved enriched gene sets from in Table 1.
Revised Sample Version

ID	Name	Molecular Function
GO:0048018	Receptor Ligand Activity	The activity of a gene product that interacts with a receptor to effect a change in the activity of the receptor. Ligands may be produced by the same cell that expresses the receptor. Ligands may also be expressed at the plasma membrane of an adjacent cell (e.g., Notch ligands) or be secreted and diffuse extracellularly from their point of origin to the receiving cell (e.g., interleukins).
GO:0030545	Signaling receptor activator activity	Binds to and modulates the activity of a receptor.
GO:0044183	Protein folding chaperone	Binding to a protein or a protein-containing complex to assist the protein folding process.
HSA-373076	Class A/1 (Rhodopsin-like receptors)	Rhodopsin-like receptors (class A/1) are the largest group of GPCRs and are the best studied group from a functional and structural point of view. They show great diversity at the sequence level and thus, can be subdivided into 19 subfamilies (Subfamily A1-19) based on a phylogenetic analysis (Joost P and Methner A, 2002). They represent members that include hormone, light and neurotransmitter receptors and encompass a wide range of functions including many autocrine, paracrine, and endocrine processes.

Supplementary Table 3. List of the 13 differentially expressed genes ($|log2FC| > 0.5$; p-adj. < 0.05) conserved across both batches, all three cell lines, and all condition comparisons, along with descriptions of their associated biological processes.

HGNC Symbol	Gene Name	Biological Process
CNTF	Ciliary Neurotrophic Factor	Promotes survival of in vitro and in vivo neuronal cell types
FGF18	Fibroblast Growth Factor 18	Involved in embryonic development, cell growth, morphogenesis, tissue repair, tumor growth, and invasion
GNRH1	Gonadotropin-Releasing Hormone 1	This gene encodes a proteolytically processed preproprotein to generate a peptide member of the gonadotropin-releasing hormone (GnRH) family of peptides.
GNRH2	Gonadotropin-Releasing Hormone 2	Codes for a preprotein, however, translation in humans has not yet been shown.
HBEGF	Heparin Binding EGF-like Growth Factor	Enables growth factor activity and heparin binding. It is located in the cell surface and extracellular space.
JAG1	Jagged canonical notched ligand 1	Human jagged 1 is the ligand for the receptor notch 1; the latter is involved in signaling processes.
LTA	Lymphotoxin Alpha	The encoded protein, a tumor necrosis factor family member, is a cytokine lymphocytes produce. The protein is highly inducible and secreted and forms heterotrimers with lymphotoxin-beta, which anchor lymphotoxin-alpha to the cell surface. This protein also mediates a large variety of inflammatory, immunostimulatory, and antiviral responses, is involved in the formation of secondary lymphoid organs during development and plays a role in apoptosis.
MIA	MIA SH3 domain containing	Predicted to enable growth factor activity. Predicted to be involved in extracellular matrix organization. Predicted to act upstream of or within cell-matrix adhesion. Predicted to be located in extracellular space
PGF	Placental Growth Factor	Enables growth factor activity. It is involved in positive regulation of cell population proliferation. It is predicted to be located in the extracellular region. Predicted to be active in extracellular space
PSPN	Persephin	This gene encodes a secreted ligand of the GDNF (glial cell line-derived neurotrophic factor) subfamily and TGF-beta (transforming growth factor-beta) superfamily of proteins. The encoded preproprotein is proteolytically processed to generate the mature protein. This protein signals through the RET receptor tyrosine kinase and a GPI-linked coreceptor and promotes the survival of neuronal populations.
SEMA4D	Semaphorin 4D	Enables identical protein binding activity, semaphorin receptor binding activity, and transmembrane signaling receptor activity. It involves several processes, including positive phosphatidylinositol 3-kinase signaling, neuron projection development regulation, and phosphate metabolic process regulation. It is an integral component of the plasma membrane.
SEMA7A	Semaphorin 7A	This gene encodes a member of the semaphorin family of proteins. The encoded preproprotein is proteolytically processed to generate the mature glycosylphosphatidylinositol (GPI)-anchored membrane glycoprotein. The encoded protein is found on activated lymphocytes and erythrocytes and may be involved in immunomodulatory and neuronal processes.

TNF	Tumor Necrosis Factor	This gene encodes a multifunctional proinflammatory cytokine that belongs to the tumor necrosis factor (TNF) superfamily. Macrophages mainly secrete this cytokine. It can bind to and thus function through its TNFRSF1A/TNFR1 and TNFRSF1B/TNFBR receptors. This cytokine regulates a broad spectrum of biological processes, including cell proliferation, differentiation, apoptosis, lipid metabolism, and coagulation.

Supplementary Table 4. Summary table of quality control for Batch 1.

Sample name	Raw reads	Clean reads	Raw bases	Clean bases	Error rate(%)	Q20(%)	Q30(%)	GC content(%)
HeLa1cnt	35872594	35392491	10.8G	10.6G	0.03	97.86	94.18	50.52
HeLa2cnt	40588307	39763626	12.2G	11.9G	0.03	97.76	93.96	50.28
HeLa3cnt	48968978	47830612	14.7G	14.3G	0.03	97.85	94.19	50.58
HeLa1_0R	31967532	31443462	9.6G	9.4G	0.03	97.69	93.84	50.66
HeLa2_0R	37072811	36192382	11.1G	10.9G	0.02	97.89	94.33	50.42
HeLa3_0R	41466226	40777276	12.4G	12.2G	0.03	97.8	94.11	50.71
HeLa1_8R	30312159	29652893	9.1G	8.9G	0.03	97.76	93.96	51.29
HeLa2_8R	35227814	34635299	10.6G	10.4G	0.03	97.86	94.2	50.45
HeLa3_8R	33843797	33328748	10.2G	10.0G	0.03	97.86	94.21	49.7
HEK1cnt	39926163	39126229	12.0G	11.7G	0.03	97.79	94.06	48.43
HEK2cnt	34041857	33416198	10.2G	10.0G	0.03	97.78	94.03	48.92
HEK3cnt	35956039	35315742	10.8G	10.6G	0.03	97.73	93.89	48.31
HEK1_0R	40629763	40099360	12.2G	12.0G	0.03	97.61	93.7	48.2
HEK2_0R	38002642	37410561	11.4G	11.2G	0.03	97.52	93.5	48.3
HEK3_0R	40373617	39155548	12.1G	11.7G	0.03	97.72	93.96	49.14
HEK1_8R	33442598	32604721	10.0G	9.8G	0.03	97.68	93.86	48.38
HEK2_8R	35327546	34572374	10.6G	10.4G	0.03	97.82	94.22	48.03
HEK3_8R	42653168	41520036	12.8G	12.5G	0.03	97.67	93.83	48.5
Hep1cnt	35856136	34755018	10.8G	10.4G	0.03	97.71	93.84	48.92
Hep2cnt	32429635	31241941	9.7G	9.4G	0.03	97.73	93.84	45.92
Hep3cnt	38344302	37715012	11.5G	11.3G	0.03	97.7	93.87	48.28
Hep1_0R	42554874	41628733	12.8G	12.5G	0.03	97.55	93.67	50.26
Hep2_0R	38212434	37586682	11.5G	11.3G	0.03	97.61	93.69	49.57
Hep3_0R	39235855	38542215	11.8G	11.6G	0.03	97.56	93.66	50.12
Hep1_8R	32004931	31142253	9.6G	9.3G	0.03	97.52	93.54	47.79
Hep2_8R	43290892	42319750	13.0G	12.7G	0.03	97.36	93.14	48.29
Hep3_8R	38439567	37525468	11.5G	11.3G	0.03	97.43	93.33	48.47

Supplementary Table 5. Summary table of quality control for Batch 2.

Sample name	Raw reads	Clean reads	Raw bases	Clean bases	Error rate (%)	Q20(%)	Q30(%)	GC content (%)
HeLa1cnt	62016498	9.3G	60845478	9.13G	0.02	98.21	94.87	49.5
HeLa2cnt	70595362	10.59G	69120548	10.37G	0.02	98.23	95.01	49.52
HeLa3cnt	60631278	9.09G	59517000	8.93G	0.02	98.23	95.13	50.53
HeLa4cnt	81419890	12.21G	80080716	12.01G	0.02	98.19	94.82	49.28
HeLa5cnt	73492284	11.02G	72283092	10.84G	0.02	98	94.8	49.95
HeLa6cnt	62844008	9.43G	61885638	9.28G	0.02	98.24	94.99	49.85
HeLa1_0R	61676822	9.25G	60913992	9.14G	0.02	98.28	95.18	50.3
HeLa2_0R	61288296	9.19G	60459190	9.07G	0.02	98.31	95.23	50.05
HeLa4_0R	63662714	9.55G	62807390	9.42G	0.02	98.31	95.24	49.68
HeLa5_0R	61124770	9.17G	60088668	9.01G	0.02	98.28	95.13	50.07
HeLa6_0R	61395698	9.21G	60252900	9.04G	0.02	98.26	95.19	50.69
HeLa1_8R	61353524	9.2G	60294048	9.04G	0.02	98.19	94.94	50.38
HeLa3_8R	60850104	9.13G	59765084	8.96G	0.02	98.25	95.16	50.11
HeLa4_8R	62198866	9.33G	60957704	9.14G	0.02	98.02	94.48	49.66
HeLa5_8R	63932420	9.59G	62589710	9.39G	0.02	98.08	94.7	50.37
HeLa6_8R	61521638	9.23G	60228370	9.03G	0.02	98.26	95.21	50.27
HEK1cnt	66272392	9.94G	65330354	9.8G	0.02	98.3	95.17	49.92
HEK2cnt	60035250	9.01G	58712308	8.81G	0.02	98.23	95.07	50.36
HEK3cnt	64145522	9.62G	63225354	9.48G	0.02	98.29	95.1	49.15
HEK4cnt	65659452	9.85G	64716964	9.71G	0.02	98.35	95.26	48.98
HEK5cnt	60758956	9.11G	59607368	8.94G	0.02	98.26	95	48.95
HEK6cnt	61580132	9.24G	60342074	9.05G	0.02	98.4	95.42	49.15
HEK1_0R	69441930	10.42G	68057050	10.21G	0.02	98.07	94.72	50.09
HEK2_0R	67217478	10.08G	65897520	9.88G	0.02	98.03	94.63	49.76
HEK3_0R	61440470	9.22G	60215412	9.03G	0.02	98.25	95.16	48.6
HEK4_0R	64208408	9.63G	62884812	9.43G	0.02	98.26	95.24	48.42
HEK5_0R	60640764	9.1G	59344610	8.9G	0.02	98.23	95.16	48.85
HEK6_0R	60250308	9.04G	59084584	8.86G	0.02	98.24	95.19	49.16
HEK1_8R	63929742	9.59G	63030320	9.45G	0.02	98.42	95.47	49.5
HEK2_8R	63883690	9.58G	62590046	9.39G	0.02	98.44	95.53	49.4
HEK3_8R	78514742	11.78G	76691164	11.5G	0.02	98.41	95.46	48.93
HEK4_8R	58987108	8.85G	57708896	8.66G	0.02	98.29	95.14	49.43
HEK5_8R	63175986	9.48G	62004916	9.3G	0.02	98.24	95.02	49.55
HEK6_8R	63239640	9.49G	62031320	9.3G	0.02	98.39	95.39	49.39
Hep1cnt	67481398	10.12G	65973190	9.9G	0.02	98.4	95.42	48.33
Hep2cnt	79463982	11.92G	77844494	11.68G	0.02	98.32	95.24	47.94
Hep3cnt	62406870	9.36G	61270232	9.19G	0.02	98.25	95.03	48.08
Hep4cnt	63241240	9.49G	61807164	9.27G	0.02	98.24	95.04	49.3

Hep5cnt	65096858	9.76G	63728104	9.56G	0.02	98.32	95.21	48.45
Hep6cnt	68099944	10.21G	66821570	10.02G	0.02	98.19	94.86	47.62
Hep1_0R	62107654	9.32G	60784062	9.12G	0.02	98.32	95.22	49.26
Hep2_0R	60533366	9.08G	59547872	8.93G	0.02	98.34	95.32	49.4
Hep3_0R	64125286	9.62G	62786732	9.42G	0.02	98.32	95.22	49.1
Hep4_0R	63549094	9.53G	62115522	9.32G	0.02	98.2	95.06	50
Hep5_0R	60337954	9.05G	59417138	8.91G	0.02	98.17	94.86	49.09
Hep6_0R	66796434	10.02G	65204242	9.78G	0.02	98.16	94.82	48.53
Hep1_8R	65094562	9.76G	63457930	9.52G	0.02	98.19	94.9	48.59
Hep2_8R	63557536	9.53G	62146416	9.32G	0.02	98.15	94.85	49.11
Hep3_8R	62153818	9.32G	61130290	9.17G	0.02	98.19	94.92	48.68
Hep4_8R	64571708	9.69G	62780996	9.42G	0.03	97.6	93.52	48.98
Hep5_8R	67192788	10.08G	65483534	9.82G	0.03	97.69	93.66	48.87
Hep6_8R	59336896	8.9G	58500944	8.78G	0.02	98.23	95.12	48.44

Supplementary Table 6. Primers used in qPCR experiments.

Classification	HGNC Symbol	Primer Sequence (5’-> 3’)	Primer Sequence (3’-5’)	Product Size (bp)
Signal Receptor Ligand Activity	LTA	ACCATTTCAGGGGTCGTCAC	GGATGGTTCAGGGAGGTGTG	131
Signal Receptor Ligand Activity	MIA	CAGGAGTGCAGCCACCCTAT	AAATAGCCCAGGCGAGCAG	194
Signal Receptor Ligand Activity	TNF	CAAGGACAGCAGAGGACCAG	TCCTTTCCAGGGGAGAGAGG	156
Heat Acclimation	HSPA1A	AGCTGGAGCAGGTGTGTAAC	CAGCAATCTTGGAAAGGCCC	154
Heat Acclimation	HSPA6	CCAGAGGAACGCCACTATCC	GGAGGGATGCCACTGAGTTC	156
Heat Acclimation	BAG3	AAGCCCAGAAGACGCACTAC	GACAGATGACCTGAACGGGG	131
Heat Acclimation	DNAJB1	GGCTCACCTGGGCTCG	CCCGGGAATTATCCCAACCC	129
Negative Control	ACTB	CTTCGCGGGCGACGAT	CCACATAGGAATCCTTCTGACC	104
Negative Control	GAPDH	CGGGAAGGAAATGAATGGGC	GGAAAAGCATCACCCGGAGG	148

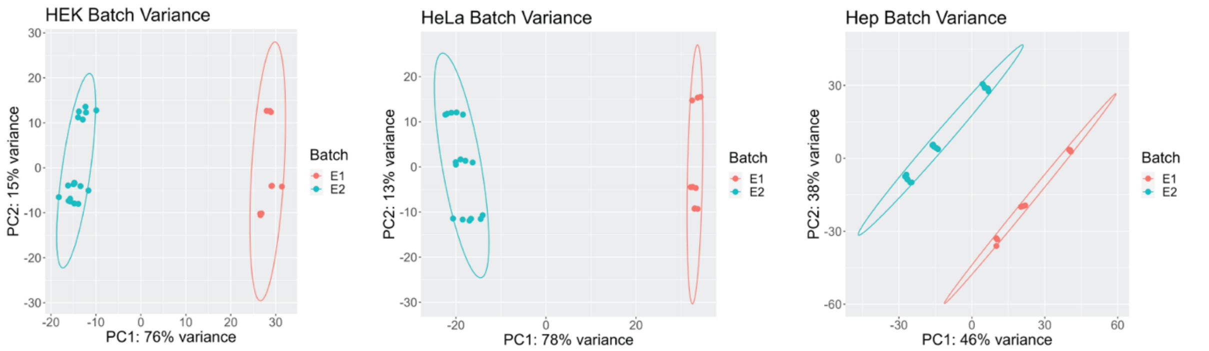

Supplementary Figure 1. PCA Analysis Highlighting Batch Effects in Cell Lines. PCA analyses of VST-normalized counts separated by cell line indicate that system variance is primarily driven by batch effects in HEK293 (Figure 1A), HeLa (Figure 1B), and HepG2 (Figure 1C) cells. The PCA explains 76%, 78%, and 46% of the variance in each cell line, respectively, demonstrating the impact of batch variability on gene expression profiles.

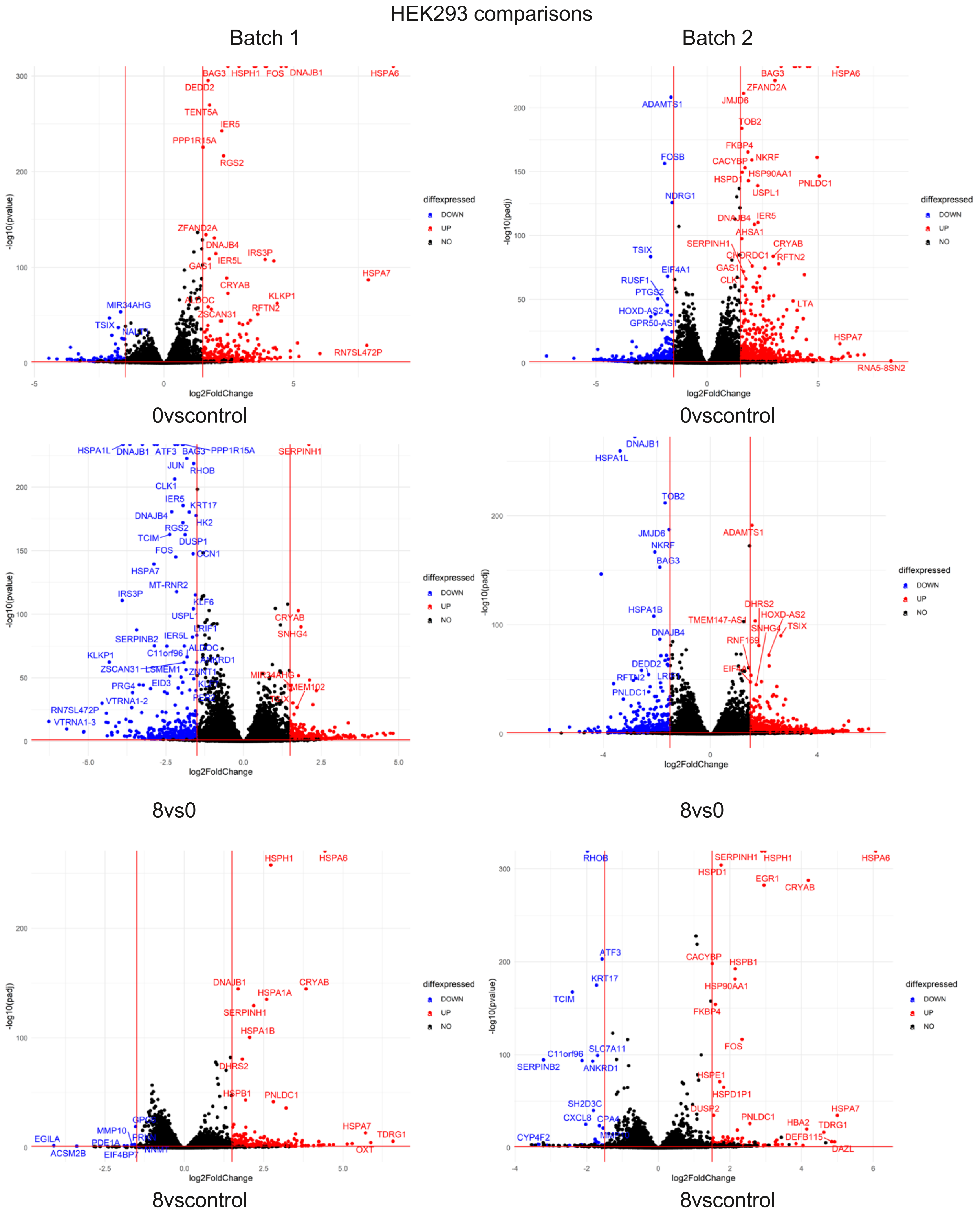

HEK293 comparisons
Batch 1
Batch 2
0vscontrol
0vscontrol
8vs0
8vs0
8vscontrol
8vscontrol
log2FoldChange
-log10(pvalue)
-log10(padj)
diffexpressed
DOWN
UP
NO
BAG3 HSPH1 FOS DNAJB1 HSPA6
DEDD2
TENT5A
IER5
PPP1R15A
RGS2
ZFAND2A
DNAJB4
IRS3P
IER5L
GAS1
HSPA7
CRYAB
KLKP1
MIR34AHG
ALDOC
RFTN2
ZSCAN31
TSIX
RN7SL472P
ADAMTS1
ZFAND2A
JMJD6
TOB2
FKBP4
FOSB
CACYBP
NKRF
HSPD1
HSP90AA1
PNLDC1
NDRG1
USPL1
IER5
SERPINH1
CRYAB
RFTN2
EIF4A1
RUSF1
PTGS2
HOXD-AS2
GPR50-AS1
LTA
RNA5-8SN2
HSPA1L
ATF3
PPP1R15A
SERPINH1
JUN
RHOB
CLK1
KRT17
HK2
TCIM
DUSP1
CCN1
MT-RNR2
KLF6
SERPINB2
C11orf96
ANKRD1
LSMEM1
ZNNT1
PRG4
EID3
VTRNA1-2
VTRNA1-3
SNHG4
TMEM102
HSPA1B
TMEM147-AS1
DHRS2
HOXD-AS2
TSIX
RNF169
DEDD2
PNLDC1
HSPA1A
HSPB1
EGILA
ACSM2B
MMP10
PDE1A
EIF4BP7
NNMT
TDRG1
OXT
EGR1
SLC7A11
SH2D3C
CXCL8
CPA4
CYP4F2
HSPE1
HSPD1P1
DUSP2
HBA2
DEFB115
DAZL

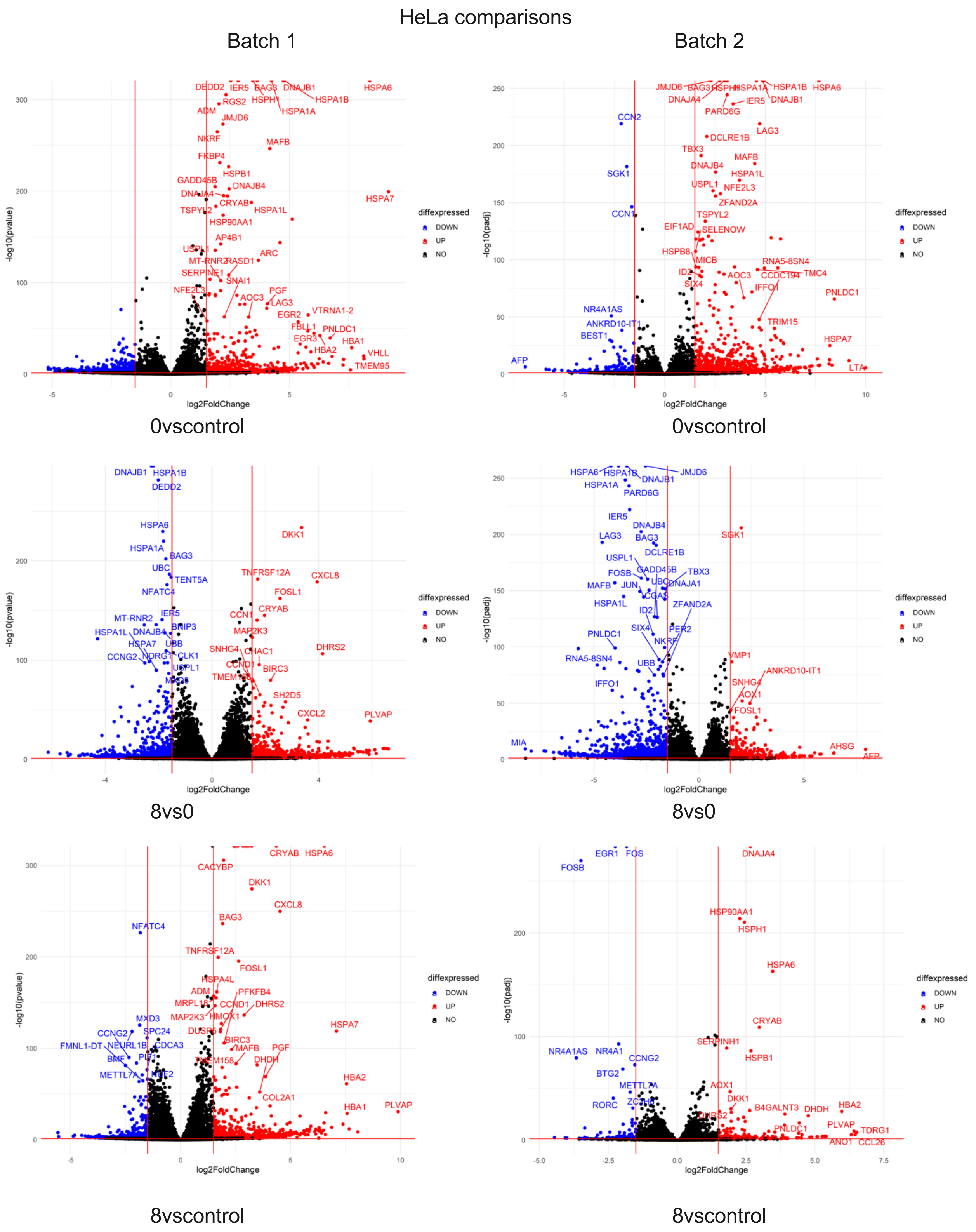

HeLa comparisons
Batch 1
Batch 2
-log10(pvalue)
-log10(padj)
log2FoldChange
diffexpressed
DOWN
UP
NO
0vscontrol
0vscontrol
8vs0
8vs0
8vscontrol
8vscontrol

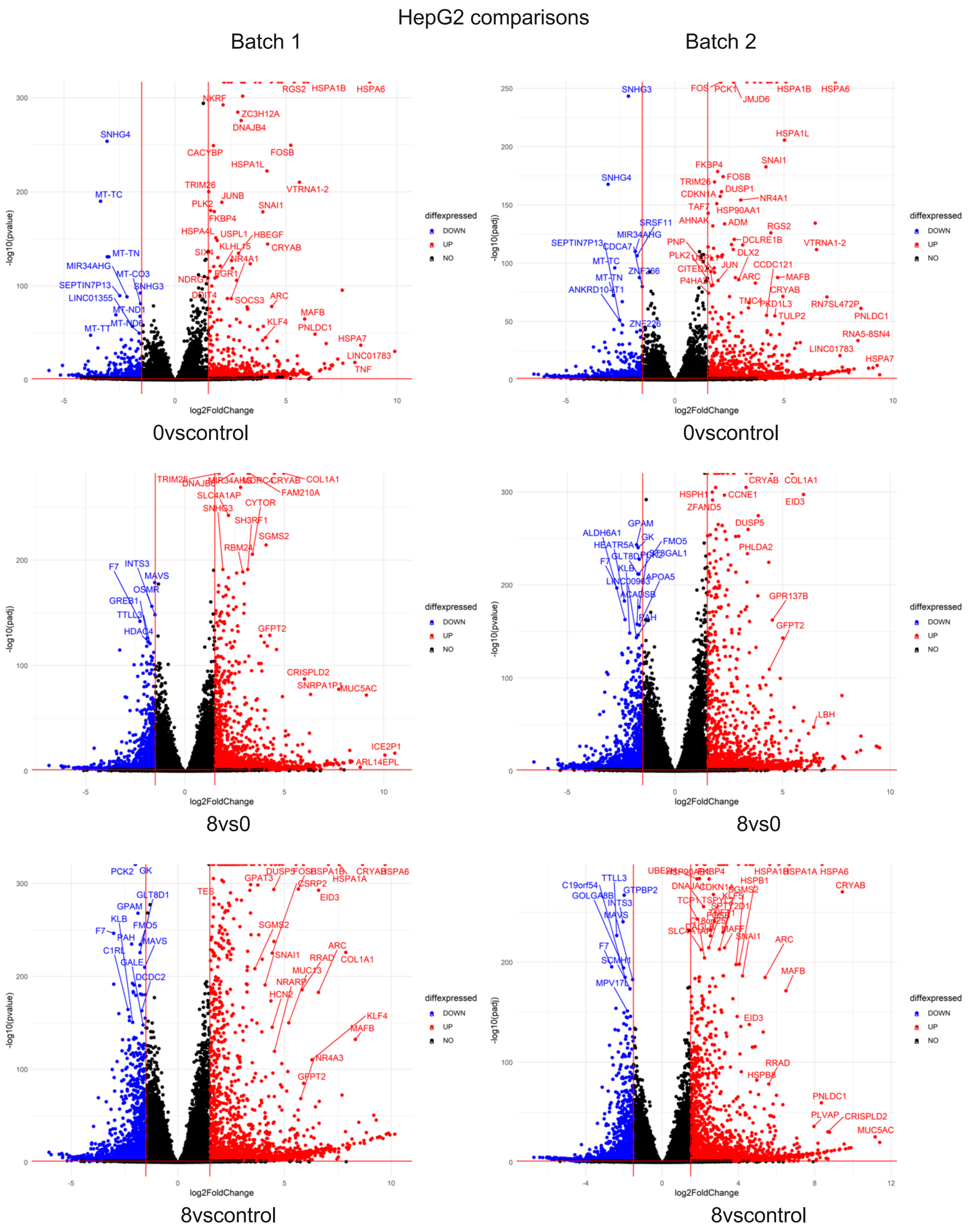

Supplementary Figure 2. Volcano Plots of Differentially Expressed Genes by Batch. Volcano plots illustrate the condition comparisons within each cell line for both Batch 1 and Batch 2. Each dot represents an individual gene, with blue dots indicating downregulated genes (log2 fold change < -1) and red dots indicating upregulated genes (log2 fold change > 1). Plots show gene expression changes for

HEK293, HeLa, and HepG2 (all comparisons for both batches). Abbreviations are as follows: 0vsControl (0 hours after heat shock vs. Control cells); 8vs0 (8 hours recovery after heat shock vs. 0 hours after heat shock cells); 8vsControl (8 hours recovery after heat shock vs. Control cells).

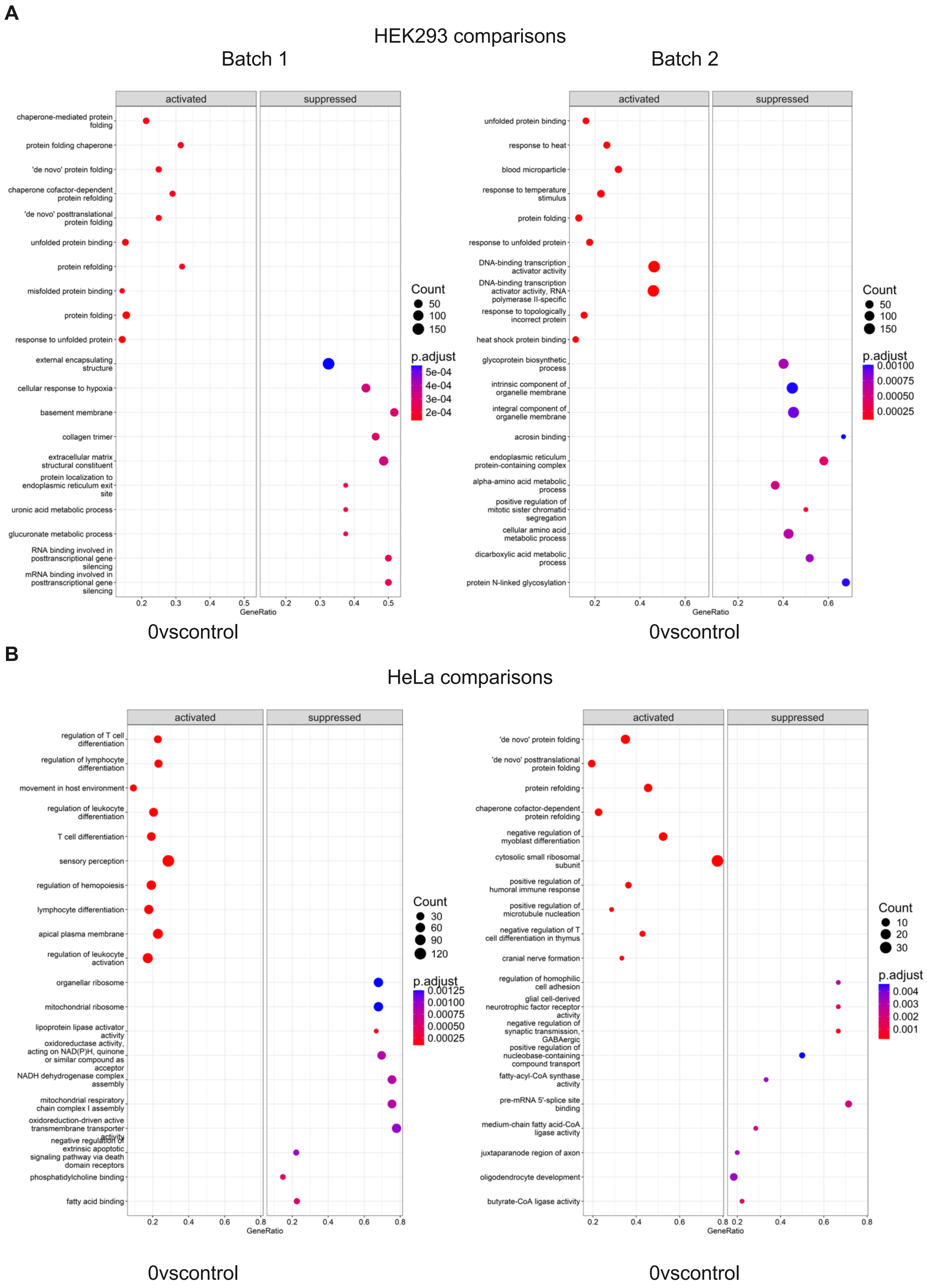
A
HEK293 comparisons
Batch 1
Batch 2
activated
suppressed
chaperone-mediated protein folding
protein folding chaperone
'de novo' protein folding
chaperone cofactor-dependent protein refolding
'de novo' posttranslational protein folding
unfolded protein binding
protein refolding
misfolded protein binding
protein folding
response to unfolded protein
external encapsulating structure
cellular response to hypoxia
basement membrane
collagen trimer
extracellular matrix structural constituent
protein localization to endoplasmic reticulum exit site
uronic acid metabolic process
glucuronate metabolic process
RNA binding involved in posttranscriptional gene silencing
mRNA binding involved in posttranscriptional gene silencing
GeneRatio
Count
50
100
150
p.adjust
5e-04
4e-04
3e-04
2e-04
0vscontrol
unfolded protein binding
response to heat
blood microparticle
response to temperature stimulus
protein folding
response to unfolded protein
DNA-binding transcription activator activity
DNA-binding transcription activator activity, RNA polymerase II-specific
response to topologically incorrect protein
heat shock protein binding
glycoprotein biosynthetic process
intrinsic component of organelle membrane
integral component of organelle membrane
acrosin binding
endoplasmic reticulum protein-containing complex
alpha-amino acid metabolic process
positive regulation of mitotic sister chromatid segregation
cellular amino acid metabolic process
dicarboxylic acid metabolic process
protein N-linked glycosylation
GeneRatio
Count
50
100
150
p.adjust
0.00100
0.00075
0.00050
0.00025
0vscontrol
B
HeLa comparisons
activated
suppressed
regulation of T cell differentiation
regulation of lymphocyte differentiation
movement in host environment
regulation of leukocyte differentiation
T cell differentiation
sensory perception
regulation of hemopoiesis
lymphocyte differentiation
apical plasma membrane
regulation of leukocyte activation
organellar ribosome
mitochondrial ribosome
lipoprotein lipase activator activity
oxidoreductase activity, acting on NAD(P)H, quinone or similar compound as acceptor
NADH dehydrogenase complex assembly
mitochondrial respiratory chain complex I assembly
oxidoreduction-driven active transmembrane transporter activity
negative regulation of extrinsic apoptotic signaling pathway via death domain receptors
phosphatidylcholine binding
fatty acid binding
GeneRatio
Count
30
60
90
120
p.adjust
0.00125
0.00100
0.00075
0.00050
0.00025
'de novo' protein folding
'de novo' posttranslational protein folding
protein refolding
chaperone cofactor-dependent protein refolding
negative regulation of myoblast differentiation
cytosolic small ribosomal subunit
positive regulation of humoral immune response
positive regulation of microtubule nucleation
negative regulation of T cell differentiation in thymus
cranial nerve formation
regulation of homophilic cell adhesion
glial cell-derived neurotrophic factor receptor activity
negative regulation of synaptic transmission, GABAergic
positive regulation of nucleobase-containing compound transport
fatty-acyl-CoA synthase activity
pre-mRNA 5'-splice site binding
medium-chain fatty acid-CoA ligase activity
juxtaparanode region of axon
oligodendrocyte development
butyrate-CoA ligase activity
GeneRatio
Count
10
20
30
p.adjust
0.004
0.003
0.002
0.001
0vscontrol
0vscontrol

C

HepG2 comparisons

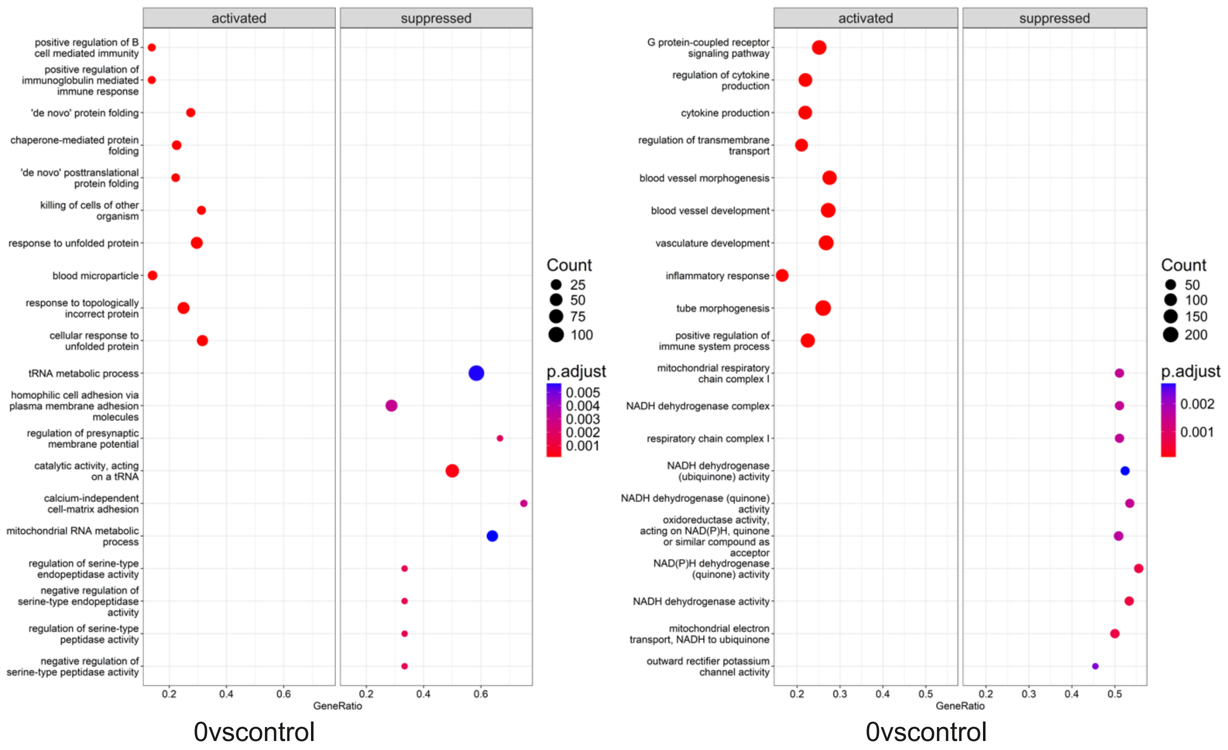

Supplementary Figure 3. Gene Set Enrichment Analysis via Dot Plots. Dot plots visualize Gene Set Enrichment Analysis (GSEA) results for the top 15 positive and top 15 negative normalized enrichment score (NES) hits [using the Human Phenotype Ontology in HEK293 (A), HeLa (B), and HepG2 (C) cell lines at 0 hours vs control cells, providing insights into enriched pathways influenced by heat shock. Abbreviations are 0vsControl (0 hours after heat shock vs. Control cells).

HEK293 comparisons

Batch 1

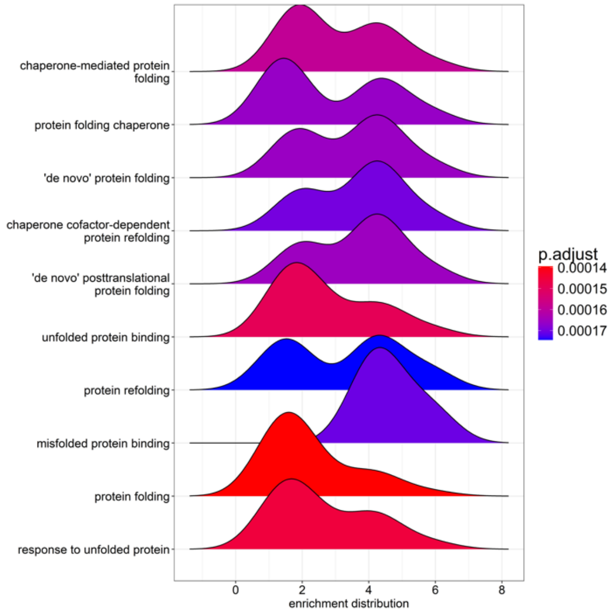

0vscontrol

Batch 2

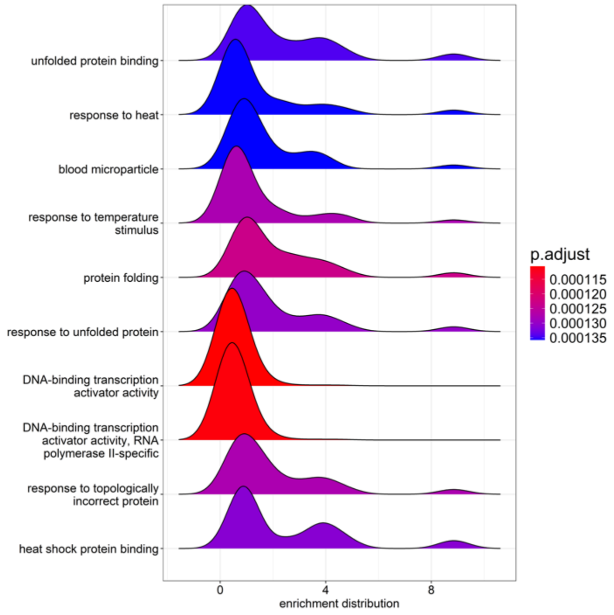

0vscontrol

HeLa comparisons

Batch 1

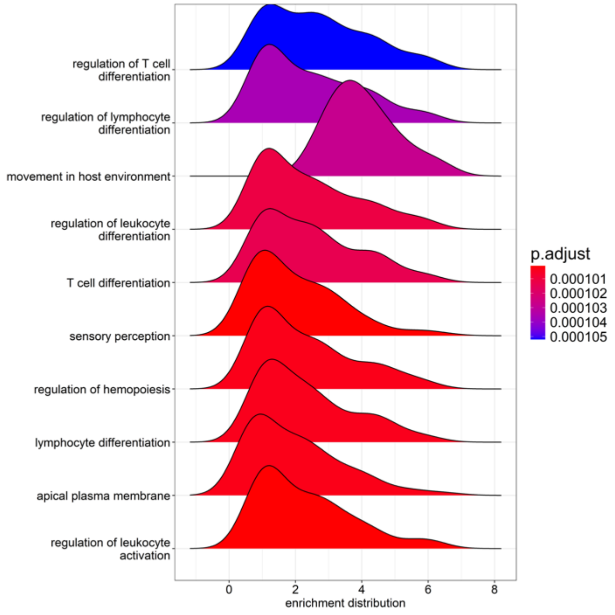

0vscontrol

Batch 2

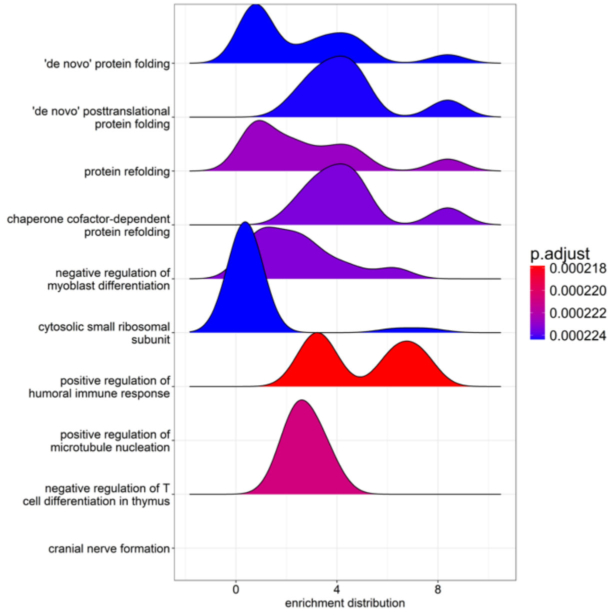

0vscontrol

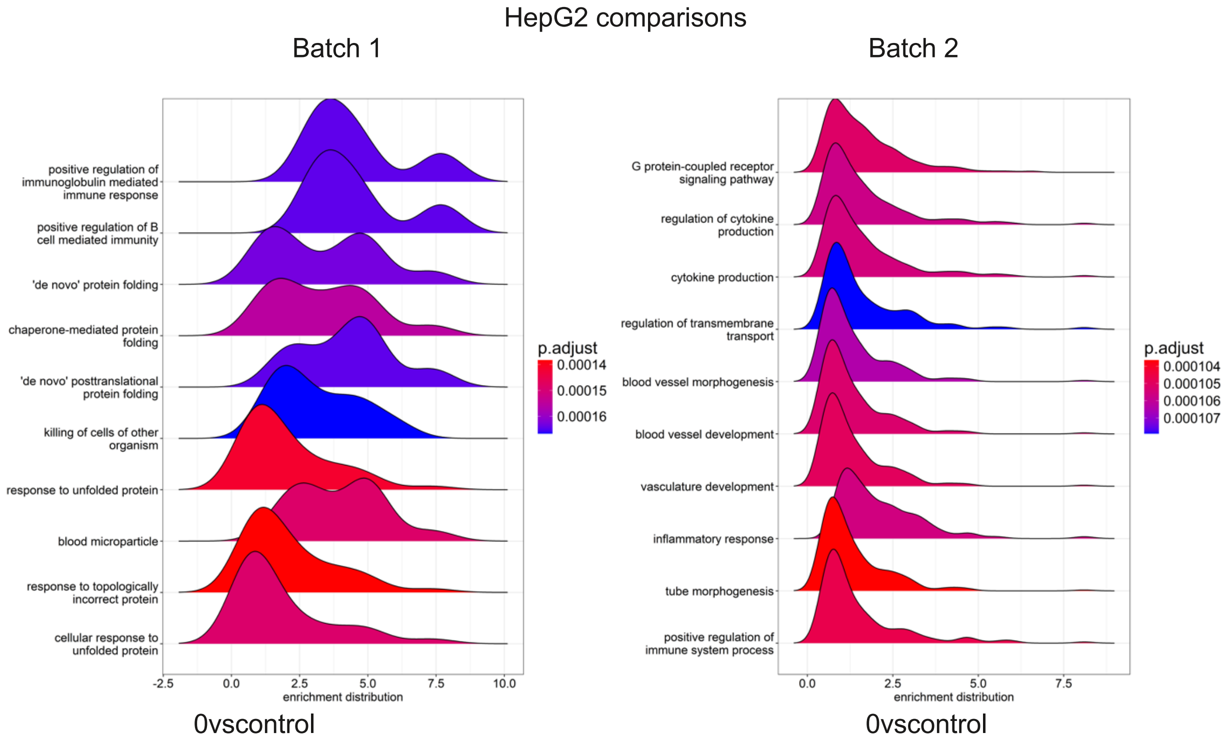

Supplementary Figure 4. Distribution of Enriched Genes Across Top GSEA Hits. Distribution plots of log2 fold changes for genes in the top 15 positively enriched GSEA pathways for HEK293, HeLa, and HepG2 cell lines at 0 hours vs. Control. Peak height indicates the number of enriched genes within each log2 fold change range. Abbreviations are as follows: 0vsControl (0 hours after heat shock vs. Control cells).

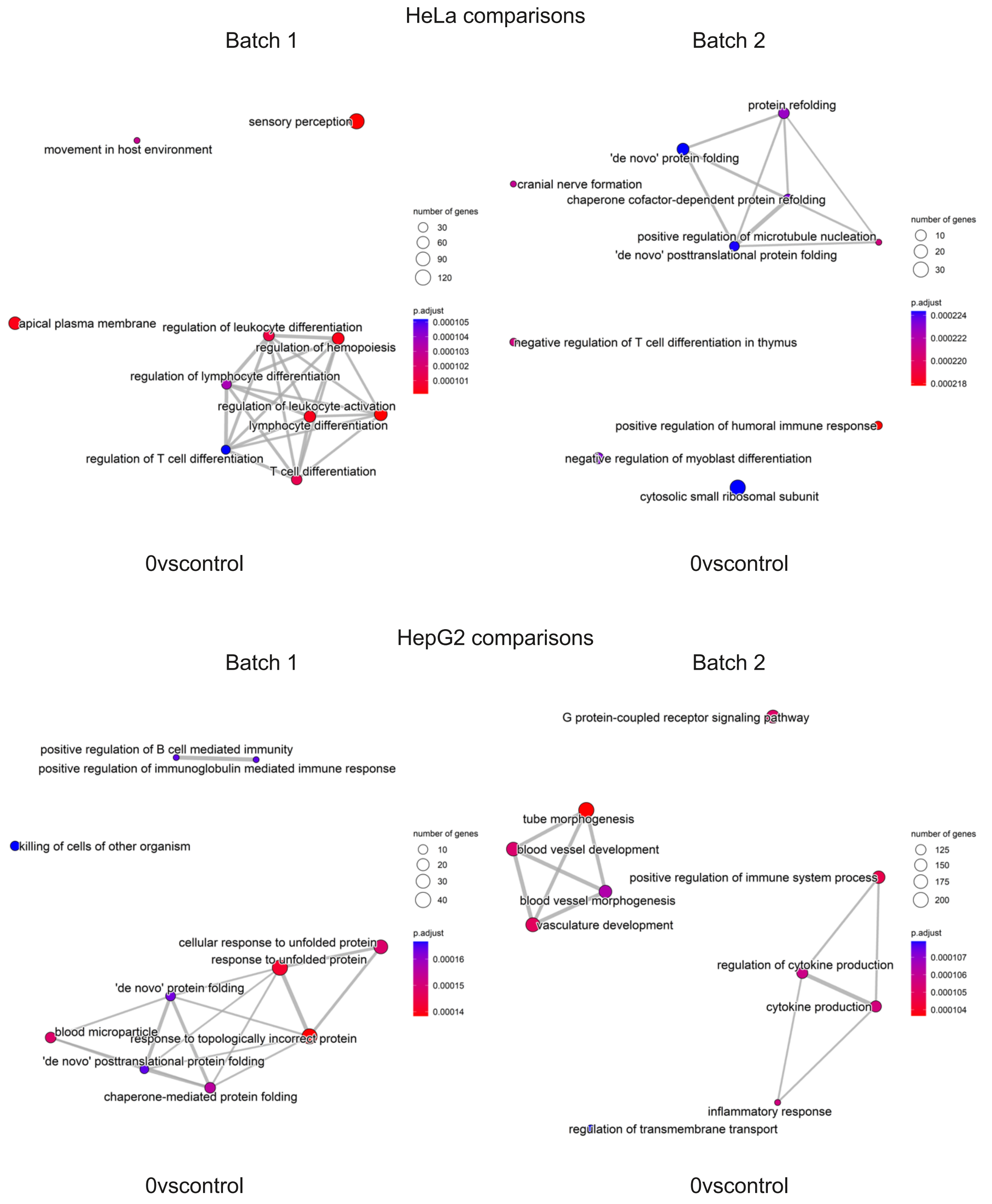

Supplementary Figure 5. Enrichment Maps of GSEA Hits for HeLa and HepG2 Cells. Network enrichment maps of the top 10 positive GSEA NES hits for HeLa and HepG2 cell lines at 0 hours vs. Control. Nodes represent enriched gene sets, while edges indicate shared genes between sets. Maps are shown for HeLa (Batch 1, top left panel; Batch 2, top right panel) and HepG2 (Batch 1, bottom left panel; Batch 2, bottom right panel), providing a systems-level perspective on pathway relationships under heat shock conditions.

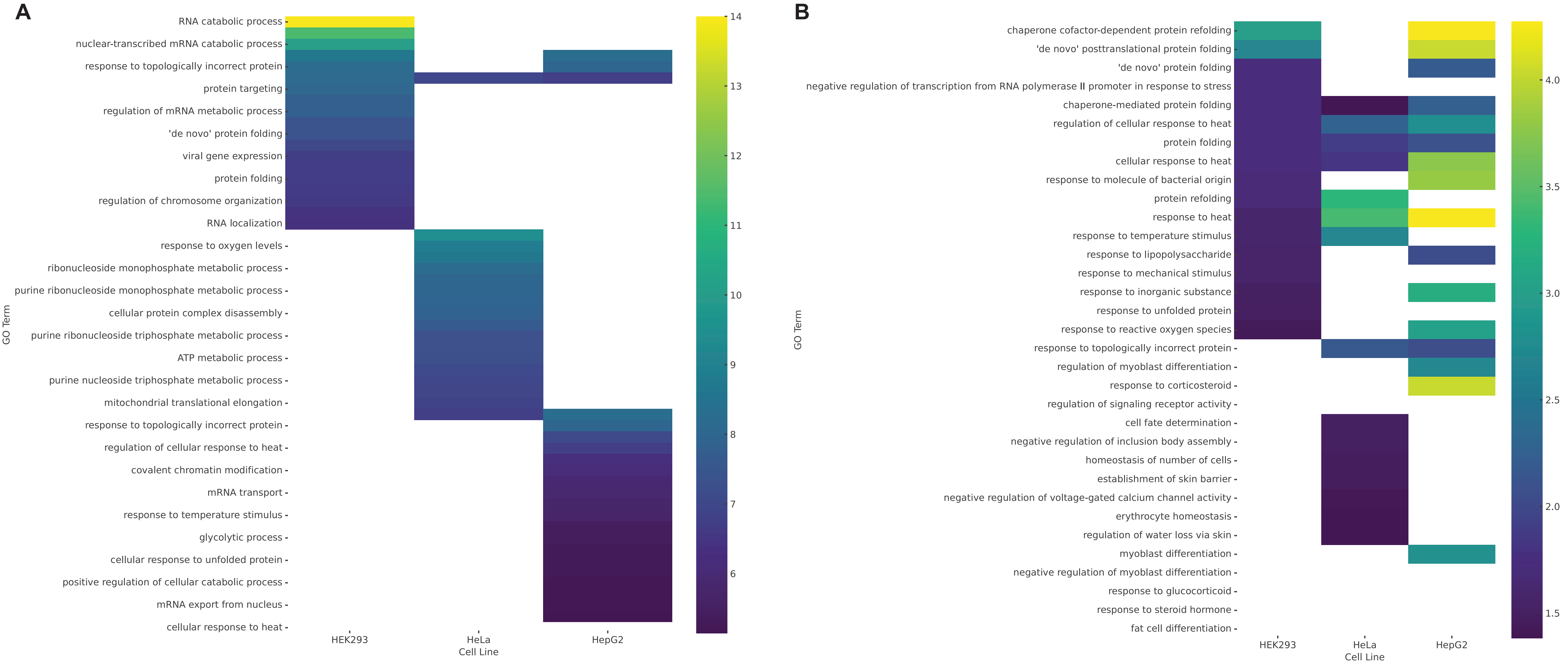
A
RNA catabolic process
nuclear-transcribed mRNA catabolic process
response to topologically incorrect protein
protein targeting
regulation of mRNA metabolic process
'de novo' protein folding
viral gene expression
protein folding
regulation of chromosome organization
RNA localization
response to oxygen levels
ribonucleoside monophosphate metabolic process
purine ribonucleoside monophosphate metabolic process
cellular protein complex disassembly
purine ribonucleoside triphosphate metabolic process
ATP metabolic process
purine nucleoside triphosphate metabolic process
mitochondrial translational elongation
response to topologically incorrect protein
regulation of cellular response to heat
covalent chromatin modification
mRNA transport
response to temperature stimulus
glycolytic process
cellular response to unfolded protein
positive regulation of cellular catabolic process
mRNA export from nucleus
cellular response to heat
GO Term
HEK293
HeLa
HepG2
Cell Line
14
13
12
11
10
9
8
7
6
B
chaperone cofactor-dependent protein refolding
'de novo' posttranslational protein folding
'de novo' protein folding
negative regulation of transcription from RNA polymerase II promoter in response to stress
chaperone-mediated protein folding
regulation of cellular response to heat
protein folding
cellular response to heat
response to molecule of bacterial origin
protein refolding
response to heat
response to temperature stimulus
response to lipopolysaccharide
response to mechanical stimulus
response to inorganic substance
response to unfolded protein
response to reactive oxygen species
response to topologically incorrect protein
regulation of myoblast differentiation
response to corticosteroid
regulation of signaling receptor activity
cell fate determination
negative regulation of inclusion body assembly
homeostasis of number of cells
establishment of skin barrier
negative regulation of voltage-gated calcium channel activity
erythrocyte homeostasis
regulation of water loss via skin
myoblast differentiation
negative regulation of myoblast differentiation
response to glucocorticoid
response to steroid hormone
fat cell differentiation
GO Term
HEK293
HeLa
HepG2
Cell Line
4.0
3.5
3.0
2.5
2.0
1.5

Supplementary Figure 6. Comparative Heatmap of -log10(p-values) for Enriched GO Terms Across HEK293, HeLa, and HepG2 Cell Lines for Batch 1 (A) and Batch 2 (B). The heatmaps depict the -log10(p-values) for the top 19 enriched Gene Ontology (GO) Biological Process (BP) terms across three cell lines: HEK293, HeLa, and HepG2. Each row corresponds to a unique GO term, and each column represents one of the cell lines. The color intensity reflects the statistical significance of enrichment, with darker colors indicating higher significance [-log10(p-value) values]. Missing values, where a GO term was not significantly enriched, are masked, and appear as blank cells in the heatmap. The complete data tables used are located in Appendix 2.

Batch 1: In HEK293, enriched GO terms are RNA catabolic process (GO:0006401); mRNA catabolic process (GO:0006402); nuclear-transcribed mRNA catabolic process (GO:0000956); response to unfolded protein (GO:0006986); response to topologically incorrect protein (GO:0035966); covalent chromatin modification (GO:0016569); protein targeting (GO:0006605); 'de novo' posttranslational protein folding (GO:0051084); regulation of mRNA metabolic process (GO:1903311); histone modification (GO:0016570); 'de novo' protein folding (GO:0006458); chaperone cofactor-dependent protein refolding (GO:0051085); viral gene expression (GO:0019080); translational initiation (GO:0006413); protein folding (GO:0006457); regulation of DNA metabolic process (GO:0051052); regulation of chromosome organization (GO:0033044); nuclear-transcribed mRNA catabolic process, nonsense-mediated decay (GO:0000184); and RNA localization (GO:0006403). In HeLa, enriched GO terms are protein-containing complex disassembly (GO:0032984); response to oxygen levels (GO:0070482); response to decreased oxygen levels (GO:0036293); ribonucleoside monophosphate metabolic process (GO:0009161); purine nucleoside monophosphate metabolic process (GO:0009126); purine ribonucleoside monophosphate metabolic process (GO:0009167); response to hypoxia (GO:0001666); cellular protein complex disassembly (GO:0043624); nucleoside monophosphate metabolic process (GO:0009123); purine ribonucleoside triphosphate metabolic process (GO:0009205); chaperone-mediated protein folding (GO:0061077); ATP metabolic process (GO:0046034); ribonucleoside triphosphate metabolic process (GO:0009199); purine nucleoside triphosphate metabolic process (GO:0009144); covalent chromatin modification (GO:0016569); nucleoside triphosphate metabolic process (GO:0009141); histone modification (GO:0016570); mitochondrial translational elongation (GO:0070125); and translational elongation (GO:0006414). In HepG2, enriched GO terms are response to topologically incorrect protein (GO:0035966); response to unfolded protein (GO:0006986); regulation of cellular response to heat (GO:1900034); histone modification (GO:0016570); covalent chromatin modification (GO:0016569); peptidyl-lysine modification (GO:0018205); mRNA transport (GO:0051028); nucleobase-containing compound transport (GO:0015931); response to temperature stimulus (GO:0009266); positive regulation of catabolic process (GO:0009896); glycolytic process (GO:0006096); interaction with host (GO:0051701); cellular response to unfolded protein (GO:0034620); ATP generation from ADP (GO:0006757); positive regulation of cellular catabolic process (GO:0031331); establishment of RNA localization (GO:0051236); mRNA export from nucleus (GO:0006406); mRNA-containing ribonucleoprotein complex export from nucleus (GO:0071427); and cellular response to heat (GO:0034605).

Batch 2: In HEK293 cells, enriched GO terms include chaperone cofactor-dependent protein refolding (GO:0051085), 'de novo' posttranslational protein folding (GO:0051084), negative regulation of transcription from RNA polymerase II promoter in response to stress (GO:0097201), regulation of cellular response to heat (GO:1900034), response to molecule of bacterial origin (GO:0002237), response to lipopolysaccharide (GO:0032496), response to reactive oxygen species (GO:0000302), and several others related to protein folding and stress responses. In HeLa cells, enriched GO terms highlight processes such as response to unfolded protein (GO:0006986), response to heat (GO:0009408), protein refolding (GO:0042026), response to topologically incorrect protein (GO:0035966), regulation of signaling receptor activity (GO:0010469), homeostasis of number of cells (GO:0048872), establishment of skin barrier (GO:0061436), negative regulation of voltage-gated calcium channel activity (GO:1901386), erythrocyte homeostasis (GO:0034101), and others involved in cellular adaptation, differentiation, and homeostasis. In HepG2 cells, enriched GO terms include response to temperature stimulus (GO:0009266), response to corticosteroid (GO:0031960), response to glucocorticoid (GO:0051384), response to steroid hormone (GO:0048545), regulation of myoblast differentiation (GO:0045661), negative regulation of myoblast differentiation (GO:0045662), fat cell differentiation (GO:0045444), and others emphasizing differentiation, hormonal response, and protein folding.

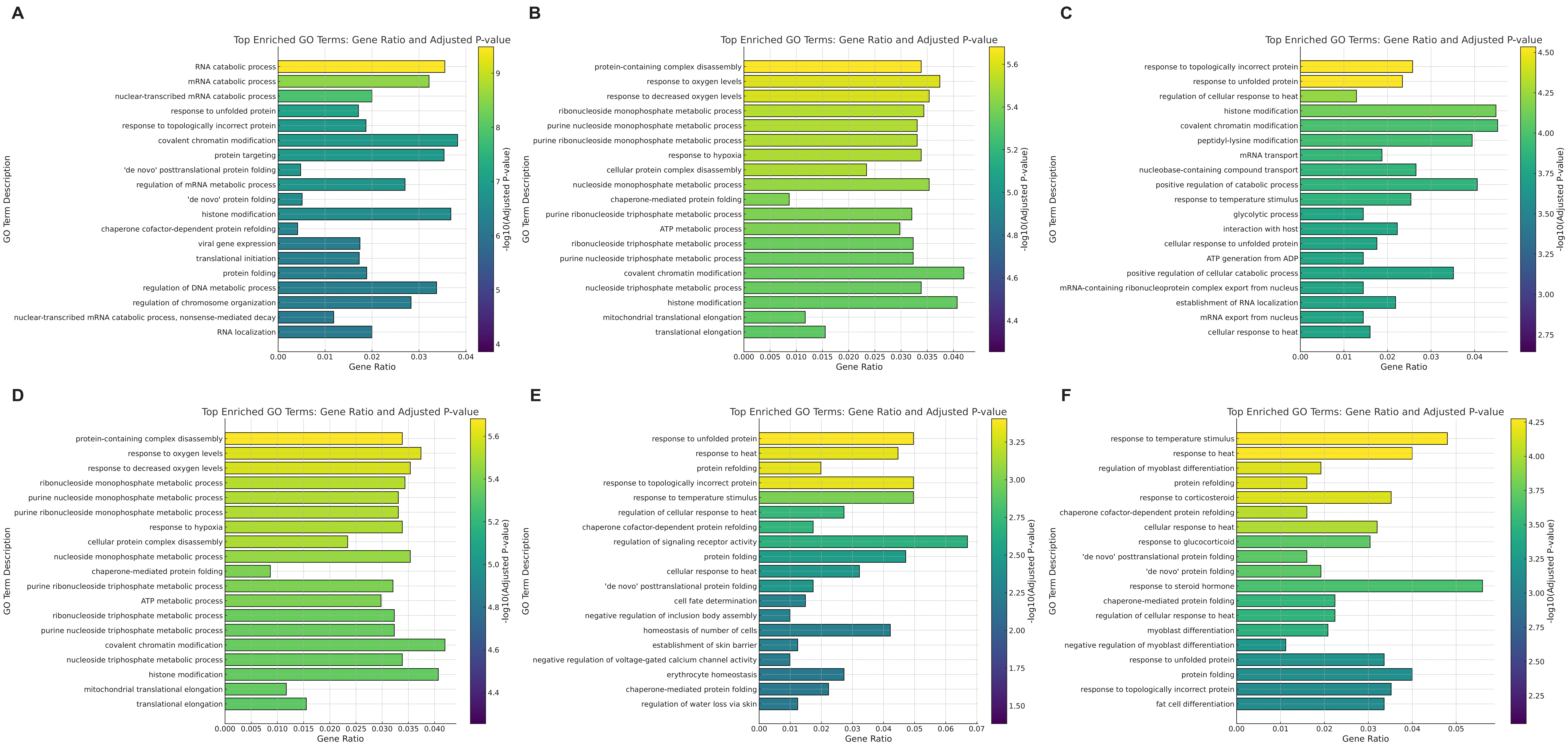
A
Top Enriched GO Terms: Gene Ratio and Adjusted P-value
GO Term Description
RNA catabolic process
mRNA catabolic process
nuclear-transcribed mRNA catabolic process
response to unfolded protein
response to topologically incorrect protein
covalent chromatin modification
protein targeting
'de novo' posttranslational protein folding
regulation of mRNA metabolic process
'de novo' protein folding
histone modification
chaperone cofactor-dependent protein refolding
viral gene expression
translational initiation
protein folding
regulation of DNA metabolic process
regulation of chromosome organization
nuclear-transcribed mRNA catabolic process, nonsense-mediated decay
RNA localization
0.00 0.01 0.02 0.03 0.04
Gene Ratio
-log10(Adjusted P-value)
4 5 6 7 8 9
B
Top Enriched GO Terms: Gene Ratio and Adjusted P-value
GO Term Description
protein-containing complex disassembly
response to oxygen levels
response to decreased oxygen levels
ribonucleoside monophosphate metabolic process
purine nucleoside monophosphate metabolic process
purine ribonucleoside monophosphate metabolic process
response to hypoxia
cellular protein complex disassembly
nucleoside monophosphate metabolic process
chaperone-mediated protein folding
purine ribonucleoside triphosphate metabolic process
ATP metabolic process
ribonucleoside triphosphate metabolic process
purine nucleoside triphosphate metabolic process
covalent chromatin modification
nucleoside triphosphate metabolic process
histone modification
mitochondrial translational elongation
translational elongation
0.000 0.005 0.010 0.015 0.020 0.025 0.030 0.035 0.040
Gene Ratio
-log10(Adjusted P-value)
4.4 4.6 4.8 5.0 5.2 5.4 5.6
C
Top Enriched GO Terms: Gene Ratio and Adjusted P-value
GO Term Description
response to topologically incorrect protein
response to unfolded protein
regulation of cellular response to heat
histone modification
covalent chromatin modification
peptidyl-lysine modification
mRNA transport
nucleobase-containing compound transport
positive regulation of catabolic process
response to temperature stimulus
glycolytic process
interaction with host
cellular response to unfolded protein
ATP generation from ADP
positive regulation of cellular catabolic process
mRNA-containing ribonucleoprotein complex export from nucleus
establishment of RNA localization
mRNA export from nucleus
cellular response to heat
0.00 0.01 0.02 0.03 0.04
Gene Ratio
-log10(Adjusted P-value)
2.75 3.00 3.25 3.50 3.75 4.00 4.25 4.50
D
Top Enriched GO Terms: Gene Ratio and Adjusted P-value
GO Term Description
protein-containing complex disassembly
response to oxygen levels
response to decreased oxygen levels
ribonucleoside monophosphate metabolic process
purine nucleoside monophosphate metabolic process
purine ribonucleoside monophosphate metabolic process
response to hypoxia
cellular protein complex disassembly
nucleoside monophosphate metabolic process
chaperone-mediated protein folding
purine ribonucleoside triphosphate metabolic process
ATP metabolic process
ribonucleoside triphosphate metabolic process
purine nucleoside triphosphate metabolic process
covalent chromatin modification
nucleoside triphosphate metabolic process
histone modification
mitochondrial translational elongation
translational elongation
0.000 0.005 0.010 0.015 0.020 0.025 0.030 0.035 0.040
Gene Ratio
-log10(Adjusted P-value)
4.4 4.6 4.8 5.0 5.2 5.4 5.6
E
Top Enriched GO Terms: Gene Ratio and Adjusted P-value
GO Term Description
response to unfolded protein
response to heat
protein refolding
response to topologically incorrect protein
response to temperature stimulus
regulation of cellular response to heat
chaperone cofactor-dependent protein refolding
regulation of signaling receptor activity
protein folding
cellular response to heat
'de novo' posttranslational protein folding
cell fate determination
negative regulation of inclusion body assembly
homeostasis of number of cells
establishment of skin barrier
negative regulation of voltage-gated calcium channel activity
erythrocyte homeostasis
chaperone-mediated protein folding
regulation of water loss via skin
0.00 0.01 0.02 0.03 0.04 0.05 0.06 0.07
Gene Ratio
-log10(Adjusted P-value)
1.50 1.75 2.00 2.25 2.50 2.75 3.00 3.25
F
Top Enriched GO Terms: Gene Ratio and Adjusted P-value
GO Term Description
response to temperature stimulus
response to heat
regulation of myoblast differentiation
protein refolding
response to corticosteroid
chaperone cofactor-dependent protein refolding
cellular response to heat
response to glucocorticoid
'de novo' posttranslational protein folding
'de novo' protein folding
response to steroid hormone
chaperone-mediated protein folding
regulation of cellular response to heat
myoblast differentiation
negative regulation of myoblast differentiation
response to unfolded protein
protein folding
response to topologically incorrect protein
fat cell differentiation
0.00 0.01 0.02 0.03 0.04 0.05
Gene Ratio
-log10(Adjusted P-value)
2.25 2.50 2.75 3.00 3.25 3.50 3.75 4.00 4.25

Supplementary Figure 7. Individual Top Enriched GO Terms after heat shock (0 h). The bar charts illustrate the top 19 enriched Gene Ontology (GO) terms in HEK293 (A, Batch 1 and D, Batch 2), HeLa (B, Batch 1 and E, Batch 2), and HepG2 (C, Batch 1 and F, Batch 2) cells after heat shock. Each bar represents a GO term, with the gene ratio (proportion of genes associated with the term) shown on the x-axis. The color intensity corresponds to the statistical significance of the enrichment, expressed as -log10(adjusted p-value), with brighter colors indicating higher significance. The data used are located in Appendix 2 and were used to generate the comparative figure (Supplementary Figure 6). The GO annotations are provided in Supplementary Figure 6.

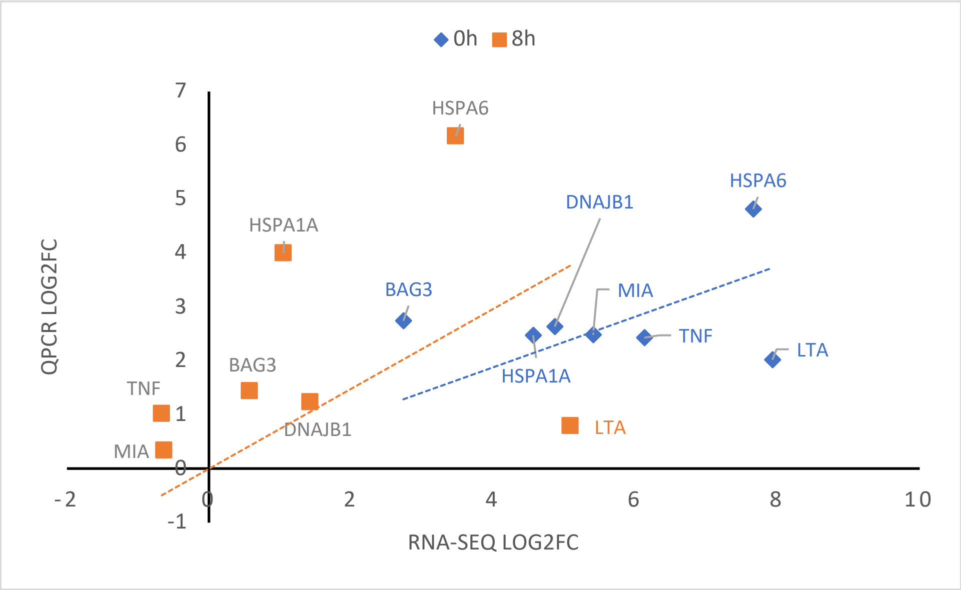

Supplementary Figure 8. Scatter plot comparing RNA-seq and qPCR fold change values. The scatter plot displays log2 fold change (log2FC) values obtained from RNA-seq (x-axis) and qPCR (y-axis) analyses for genes HSPA1A, HSPA6, BAG3, DNAJB1, LTA, MIA, and TNF at 0 hours (blue circles) and 8 hours (orange squares) post-heat shock. Data points near the diagonal indicate strong agreement between RNA-seq and qPCR measurements, while points further from the diagonal represent differences in the magnitude of fold change between the two methods, suggesting potential differences in sensitivity or dynamic range.

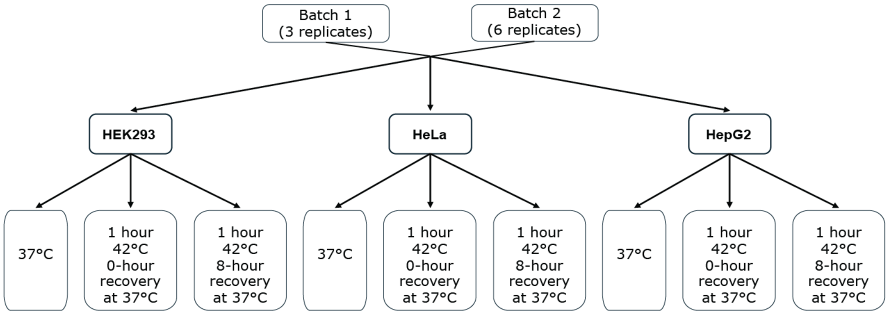

Supplementary Figure 9. Flowchart diagram illustrating the experimental design and treatment conditions. Two experimental batches were analyzed, consisting of 3 and 6 replicates for HEK293, HeLa, and HepG2 cell lines. Samples were subjected to a 1-hour mild heat shock at 42°C and allowed to recover for either 0 hours or 8 hours at 37°C. Control samples were maintained at 37°C for the entire experiment. This experimental design ensures a robust comparison of gene expression changes induced by heat shock across replicates and batches.
